# Combined Drought and Heat Stress Influences the Root Water Relation and Determine the Dry Root Rot Disease Development Under Field Conditions: A Study Using Contrasting Chickpea Genotypes

**DOI:** 10.3389/fpls.2022.890551

**Published:** 2022-05-09

**Authors:** Aswin Reddy Chilakala, Komal Vitthalrao Mali, Vadivelmurugan Irulappan, Basavanagouda S. Patil, Prachi Pandey, Krishnappa Rangappa, Venkategowda Ramegowda, M. Nagaraj Kumar, Chandra Obul Reddy Puli, Basavaiah Mohan-Raju, Muthappa Senthil-Kumar

**Affiliations:** ^1^National Institute of Plant Genome Research, Aruna Asaf Ali Marg, New Delhi, India; ^2^ICAR-Indian Agricultural Research Institute (IARI), Regional Research Center, Dharwad, India; ^3^Division of Crop Sciences, ICAR Research Complex for North Eastern Hill Region, Meghalaya, India; ^4^Department of Crop Physiology, College of Agriculture, University of Agricultural Sciences, Bengaluru, India; ^5^Division of Plant Physiology, ICAR-Indian Agricultural Research Institute, New Delhi, India; ^6^Department of Botany, Yogi Vemana University, Kadapa, India

**Keywords:** *Macrophomina phaseolina*, *Cicer arietinum*, plant water status, drought, heat, combined stress, stress tolerance, disease resistance

## Abstract

Abiotic stressors such as drought and heat predispose chickpea plants to pathogens of key importance leading to significant crop loss under field conditions. In this study, we have investigated the influence of drought and high temperature on the incidence and severity of dry root rot disease (caused by *Macrophomina phaseolina)* in chickpea, under extensive on- and off-season field trials and greenhouse conditions. We explored the association between drought tolerance and dry root rot resistance in two chickpea genotypes, ICC 4958 and JG 62, with contrasting resistance to dry root rot. In addition, we extensively analyzed various patho-morphological and root architecture traits altered by combined stresses under field and greenhouse conditions in these genotypes. We further observed the role of edaphic factors in dry root rot incidence under field conditions. Altogether, our results suggest a strong negative correlation between the plant water relations and dry root rot severity in chickpeas, indicating an association between drought tolerance and dry root rot resistance. Additionally, the significant role of heat stress in altering the dynamics of dry root rot and the importance of combinatorial screening of chickpea germplasm for dry root rot resistance, drought, and heat stress have been revealed.

## Introduction

Dry root rot (DRR), caused by the necrotrophic fungal pathogen *Macrophomina phaseolina* (anamorph, formerly referred to as *Rhizoctonia bataticola*), is one of the devastating soil-borne diseases of chickpea whose severity and incidence has increased in the recent past due to climate change (Sharma and Pande, 2013; Sinha et al., 2019, 2021). Root tissue water status and water potential, gas exchange at the root zones, and the host’s nutritional status are known to determine the incidence of DRR (Rai et al., 2022). Furthermore, at the physiological level, reduction in photosynthesis due to drought stress also predisposes the host plants to the disease (Cabral et al., 2020). However, the studies exploring drought-induced predisposition of chickpeas to dry root rot, highlighting the stress influence on plant physiology and metabolism are limited. Nevertheless, some evidence suggests a significant connection between leaf water potential and disease resistance. For instance, common bean cultivars with higher leaf water potential under drought stress showed resistance to root rot pathogens and vice versa (Mayek-Pérez et al., 2002). In addition, drought-stressed common bean plants exhibiting high stomatal resistance showed severe charcoal rot symptoms, which further indicated a high negative correlation between the disease severity, and the physiological and growth-related traits (Mayek-Pérez et al., 2002).

The role of temperature in influencing the severity of root diseases has been extensively investigated. For instance, melon accessions resistant at low temperatures were found to be susceptible to charcoal rot disease at high temperatures (de Sousa Linhares et al., 2020). Better adaptability of the pathogen to elevated temperature coupled with heat-mediated reduction of plant resistance could be the possible reasons for enhanced susceptibility to root pathogens under high temperatures. A study involving the growth of diverse isolates of *M. phaseolina* under different temperatures revealed that 35°C was the optimum temperature for its growth (Srinivas et al., 2017).

Here, we hypothesize the potential involvement of root water relations and morphological traits in affecting *M. phaseolina’s* interactions with *Cicer arietinum* (chickpea). We studied dry root rot disease development in two chickpea genotypes, ICC 4958 and JG 62 under drought and high-temperature conditions to address this hypothesis. ICC 4958 was chosen as it is one of the 1,500 diverse germplasms that showed significant drought resistance under field screening (Deokar et al., 2011). Further, the capacity of ICC 4958 to withstand terminal drought was attributed to its root growth characteristics (Kashiwagi et al., 2006). On the other hand, JG 62 (aka. ICC 4951) has been reported as a highly susceptible genotype to dry root rot resistance, exhibiting more than 90% mortality under dry root rot infection (Jayalakshmi et al., 2008; Talekar et al., 2021). Overall, using thorough systematic observations of physiological and patho-morphological characteristics under field and laboratory conditions, our study reports an interesting link between the alterations in plant water relations due to drought and temperature stress and chickpea’s response to *M. phaseolina*. In addition, edaphic factors were also studied for their influence on the DRR incidence under field conditions.

## Materials and Methods

### Plant Material

Two chickpea genotypes *viz.*, ICC 4958 and JG 62 (aka. ICC 4951) were obtained from the Indian Agricultural Research Institute, New Delhi, and were used as plant materials in all the experiments conducted in this study. The morpho-phenological characteristics of the genotypes are given in Supplementary Table 1.

### Field Trials

Field experiments, to understand the influence of root-water relations on dry root rot disease incidence were conducted as “on-season” and “off-season” field trials following randomized complete block design as described earlier (Sinha et al., 2019). Line sowing with a spacing of 10 cm × 30 cm (row-to-row × plant-to-plant) was practiced in both the field trials.

#### On-Seasonal Field Trial

A group of six diverse natural hotspot sites with low to high DRR pathogen inoculum load in the soil was selected during the year 2020–2021 (rabi season, October–March) (Irulappan and Senthil-Kumar, 2021; Supplementary Figure 1). The details of weather conditions for the experimental locations are given in Supplementary Tables 2, 3 and Supplementary Figure 2. The experiment was performed during the chickpea cultivation period specific to the location. Details related to the maintenance of the field trial experiments at the six locations are given in Supplementary Table 3. The experiment included four different treatments *viz.*, mild pathogen, a mild pathogen with drought, severe pathogen, and combined severe pathogen with drought (Supplementary Figure 3). At location 1, control, drought, and heat treatments were maintained separately in isolated field conditions, and the remaining four treatments were maintained in the pathogen-infected plots. The details of the experimental design, field layout, and stress treatments are presented in Supplementary Figure 4. Drought stress was imposed on the field plots by regulating the irrigation interval across the treatments, and soil moisture of the field locations was measured using Lutron PMS-714 soil moisture meter (Lutron Electronic Enterprise Co., Ltd., Taipei, Taiwan) to ascertain the imposition of drought (Supplementary Figure 5).

#### Off-Season Field Trial

The off-season trial during summer 2021 was conducted at field plots of the National Institute of Plant Genome Research, New Delhi (location 1). The mean atmospheric and soil temperature was considered as heat stress (Supplementary Figure 2G). Thus, the four treatments included were, heat with a mild pathogen, heat with mild pathogen and drought, heat with a severe pathogen, and heat with severe pathogen stresses and drought. Supplementary Figure 4 describes the experimental treatments and imposition of different stresses. Supplementary Figure 6 shows the imposition of drought through dynamics in soil moisture during the trial.

### Plant Growth Conditions for Pot Experiment

#### Growth Conditions

Chickpea seeds sterilized with 2% sodium hypochlorite were used for the experiments. Plants were grown under greenhouse conditions with a photoperiod of 16 h/8 h, light/dark cycle, the light intensity of 150 mmol m^–2^ s^–1^, and relative humidity of 52 ± 2% as described earlier in Sinha et al. (2019). For heat stress treatment, the temperature maintained was 37/25°C (day/night). The normal temperature maintained was 22/10°C (day/night).

#### Stress Treatments

Eight treatments viz., control, drought, pathogen, drought with pathogen, heat, heat with drought, heat with a pathogen, and heat and drought with pathogen were considered for the pot experiments. The experimental design includes a complete factorial design. The drought was imposed by with-holding the irrigation during the vegetative stage of the plant as described earlier in Sinha et al. (2019). The sterile soil rite was added with an inoculum of *M. phaseolina* isolate- Dharwad (NCBI ID OM674331) for pathogen stress. The pathogen was isolated from a small part of surface-sterilized fungal-infected root tissue taken from plant samples from Location 3 (Supplementary Figure 7). The inoculum was prepared as described by Irulappan and Senthil-Kumar (2021). An inoculum of 5:100 (weight/weight) ratio of sick culture to dry soil rite was used. Relative water content, root water potential, and disease incidence parameters were measured.

#### Control Treatments

Under field conditions, the absolute control plots were maintained at a distant location and the data from these plots were not used in this manuscript. For all analysis the mild and severe pathogen infected plots were only used as indicated in the graphs. In green house conditions, plants growing under well-watered conditions in sterile pot mix served as a control.

#### Drought Stress Treatments

Drought stress in field and pot experiments was imposed by withholding irrigation. The soil moisture of the field locations was measured using Lutron PMS-714 soil moisture meter as described above. A field capacity of 40–60% was maintained as drought stress.

#### Pathogen Stress Treatments

Pathogen stress in the field plots was imposed by growing chickpea plants in plots enriched with DRR inoculum naturally. In greenhouse conditions, the plants were infected with the pathogen using the sick pot method (Irulappan and Senthil-Kumar, 2021). For SEM studies, plants were infected using the blotting paper technique (Irulappan and Senthil-Kumar, 2021).

#### Combined Stress Treatments

For the on season field trial experiments, for combined stress treatments plants were grown in plots with less frequent irrigation to maintain 50% FC. The soil was not treated with any fungicide. In the off season field trial experiments, similar treatment served as combined stress with additional component of heat stress imposed due to the high day temperature. Under greenhouse conditions, sick soil with less frequent irrigation under ambient temperature (combined drought and pathogen) and high temperature (combined drought, heat and pathogen stress) served as combined stress treatment. The details of all the treatments are summarized in Supplementary Table 4.

### Measurement of Relative Water Content

Relative water content (RWC) from both roots and leaves was measured according to the protocol described earlier (González and González-Vilar, 2001; Sinha et al., 2019). Root and leaf samples from all the treatment plots were collected and their weights, recorded instantaneously, constituted the fresh weight. Then the samples were hydrated by placing them in de-ionized water and incubating on ice for 4 h. The tissues were weighed thereafter to measure the turgid weight. The samples were then oven-dried for 48 h at a temperature of 60° to measure the dry weight. Percentage RWC of the samples (roots and leaves) was calculated using the following formula:


$$Rootrelativewatercontent\left(\%\right)=\frac{Freshtissueweight-Drytissueweight}{Turgidtissueweight-Drytissueweight}\times 100$$

### Soil, Root, and Leaf Water Potential Quantification

Soil, root, and leaf samples were collected in 1.5 ml microcentrifuge tubes. The sample tubes were sealed with surgical tapes. The samples were then stored at −80°C till further processing. The uniformly kneaded soil samples or the cell sap from frozen leaf or root samples were used for water potential measurements. The cell sap was extracted from root and leaf samples by crushing followed by centrifugation at 12,000 rpm for 1 min. Then, the cell sap/soil samples were analyzed for water potential using a Psypro system with C52 sample chambers (Wescor PSYPRO Automated Soil and Leaf Water Potential System, United States) (Kumar et al., 2012).

### Root System Architecture Measurement

The plants were grown in pots (24 cm × 15 cm length and diameter). Pathogen and drought stress was imposed in the same way as described above. Forty-five days old plants were uprooted and washed with RO water. The clean roots were then scanned using the Scanjet G4050 Photo Scanner (Hewlett-Packard, New Delhi). Image analysis was done using GiA Roots to acquire root architecture traits like network area, length, and volume (Galkovskyi et al., 2012).

### Scanning Electron Microscope

Microsclerotia-infected plant root samples were sampled in intervals starting from 1 DAI to 5 DAI (Sinha et al., 2019; Irulappan and Senthil-Kumar, 2021). The samples were treated with 2.5% glutaraldehyde in 0.1 M sodium phosphate buffer (pH 7.2) later they are vacuum infiltrated. The roots were then dehydrated twice in acetone (30, 50, 70, 80, 90, 95, and 100%) for 10 mins each time in each percentage. The roots were maintained in 100% acetone. A critical point dryer (Leica EM CPD300, Leica Biosystems, Mumbai, India) was used to dry the critical points. Scanning electron microscopy (SEM, Carl Zeiss-Strasse, Oberkochen, Germany) was used to take pictures at 100×, 250×, and 500× magnifications.

### Evaluation of Microsclerotial Attachment in Roots

Microsclerotia-infected and well-watered plant root samples from the two genotypes were subjected to SEM as described above. The plants were infected using the blotting paper method (Irulappan and Senthil-Kumar, 2021). The number of attached microsclerotia clearly visible in the images were manually counted to estimate the extent of microslerotial attachment in the two genotypes (Irulappan et al., 2022).

### Disease Identification and Assessment of Disease Incidence and Disease Index

Dry root rot disease was recognized based on its characteristic symptoms *viz.*, straw-colored dry leaves, taproot devoid of lateral roots, and presence of dark-colored microsclerotia in split open roots. Etiology was confirmed by the PCR method (Irulappan and Senthil-Kumar, 2021). Percent disease incidence (DI) was calculated in each treatment plot using the following formula:


$$Diseaseincidence\left(\%\right)=\frac{Numberofinfectedplantsinatreatmentplot}{Totalnumberofplantspresentinthattreatmentplot}\times 100$$

The disease index was calculated as described earlier (Sinha et al., 2019). The disease score for calculating the disease severity for both pot and field experiments was assigned using the representative symptom score panel given in Supplementary Figure 8.

### Statistical Analysis

All field and greenhouse data were analyzed by calculating averages from replicates of each treatment for each parameter and were tested using two-way ANOVA analysis using Tukey’s posthoc test. Associations among the disease incidence, weather data, and important edaphic factors were tested using the Pearson correlation using IBM^®^ SPSS^®^ Statistics 26.0 (or) 14^1^. The Pearson’s correlation coefficient was used to test the relationship among the test treatments in the current study.

## Results

### Reduction in Plant Water Status Increases the Disease Incidence

**FIGURE 1:**
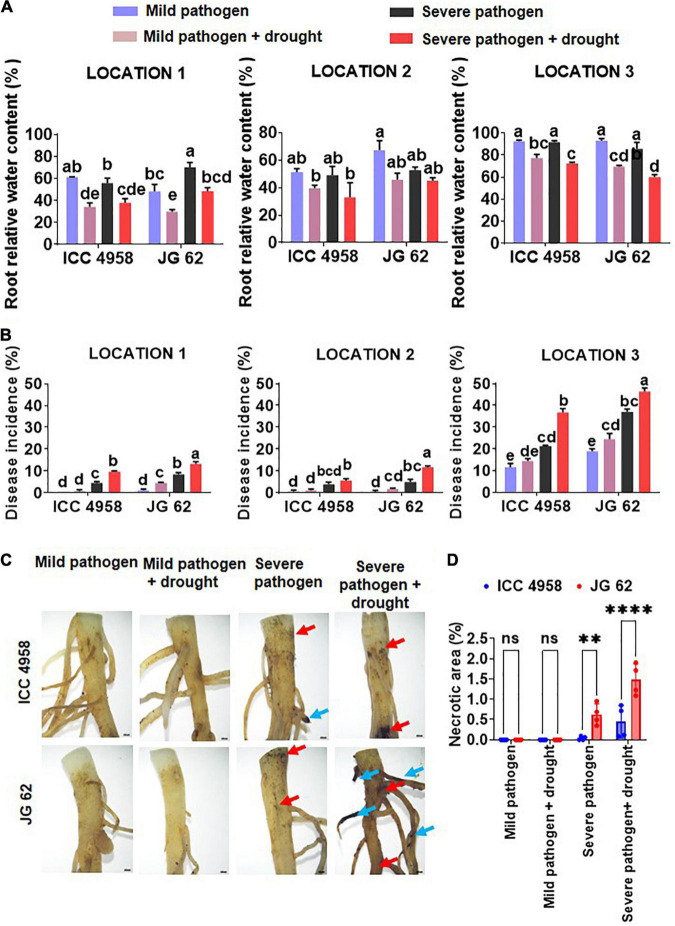
On-season field trials exhibiting the influence of plant-water relations in dry root rot (DRR) disease aggravation. The four treatments considered were- mild pathogen (fields frequent irrigated to maintain 80% FC and treated with appropriate fungicide), mild pathogen + drought (less frequently irrigated fields to maintain 50% FC and treated with appropriate fungicide), severe pathogen (frequently irrigated fields to maintain 80% FC without any fungicide application), severe pathogen + drought (less frequently irrigated fields to maintain 50% FC without any fungicide application) for both the genotypes ICC 4958 and JG 62. **(A)** Root relative water content of the two contrasting chickpea genotypes under different stress and their combinations across different experimental locations. The bars in the following graphs are the averages of their respective block replicates with standard error as error bars. Under field conditions, we did not observe a non-symptomatic control plot as the minor infection was reported in the control (fungicide treated) plot as well. This treatment is mentioned as a “mild pathogen”. **(B)** The disease incidence data, was collected at the late podding stage and additionally at the harvest stage from plants with symptomatic roots for confirmation from three locations under different stress treatments. The bars in the following graphs are the averages of their respective block replicates with standard error as error bars. **(C)** Root images showing the root branching zone 1 of 29 days old JG 62 and ICC 4958 plants exposed to a mild pathogen, severe pathogen, mild pathogen + drought, severe pathogen + drought, for 10 days under greenhouse conditions. The dark-colored lesions show the severity and higher infection in JG 62 over ICC 4958. Root images were captured under 0.63X objective lens of SMZ25 research stereomicroscope. Blue arrows show the necrotized lateral roots. Red arrows indicate the necrotic spots. The details of the locations are provided in Supplementary Table 2. Statistical significance between means was checked by two-way ANOVA and Tukey’s Posthoc test. The different letters denote a significant difference between mean at *p* < 0.05. **(D)** Graph represents the root necrosis area (%) of two cultivars for different treatments mild pathogen, severe pathogen, mild pathogen + drought, severe pathogen + drought. The bars on the graph indicate the averages of different treatments for 4 replicates with standard error as an error bar. Statistical significance difference between means is checked by two-way ANOVA and sidak’s mean multiple comparison test. The ^**^p < 0.01, ^****^p < 0.0001., ns, non-significant.

Incoherence with our previous observations (Supplementary Figure 9; Sinha et al., 2019), we found that drought predisposes both the genotypes to DRR irrespective of their level of drought tolerance. The combined drought and DRR infection were also found to be more deleterious to the overall plant performance and caused a maximum reduction in the yield (Supplementary Figure 10). For instance, yield reductions of about 57.35 and 66.99% were observed under combined drought and severe pathogen stress in ICC 4958 and JG62 at location 1. Whereas, in severe pathogen stress-only treatments 30.35 and 24.09% yield reductions were observed in ICC 4958 and JG62, respectively. However, the trend was not statistically significant in some locations presumably due to environmental variations. Additionally, disease incidence (DI) observations across the different on-season field trial locations showed that the DRR incidence in ICC 4958 was less prominent than JG 62 under severe pathogen stress treatments with or without drought (Supplementary Figures 11, 12). Drought stress enhanced the susceptibility of JG 62 to DRR more significantly than ICC 4958, which was indicated by the notable difference between the DI of the two genotypes under drought conditions (Figure 1B). We compared the effect of individual and combined drought and DRR infection on plant water status at various stages of development in the two genotypes (Supplementary Figure 13A). Global transcriptome profile data (Irulappan et al., 2022) was analyzed to study the molecular changes occurring under combined drought and DRR infection in chickpea. We observed differential regulation of genes related to water transport, root architecture, xylem modification (lignin deposition), and hormone response (auxin, ABA, and JA) under drought, pathogen, drought and pathogen stress treatments (Supplementary Figure 13B). On average, we observed that both drought and pathogen infection negatively affected the plant water status (Figure 1A), and the DI under combined stress was more in JG 62 as compared to ICC 4958 (Figure 1B). The observation was further corroborated by the microscopic observation of JG 62 roots that showed a greater number of *M. phaseolina* induced necrotic lesions and percent necrotic area than ICC 4958 (Figures 1C,D). Thus, our experiments suggest a tissue and/or stage-specific relation between the plant water status and DRR incidence in chickpea plants.

### Drought Tolerant Genotype Displayed Reduced Severity to Dry Root Rot Disease

**FIGURE 2:**
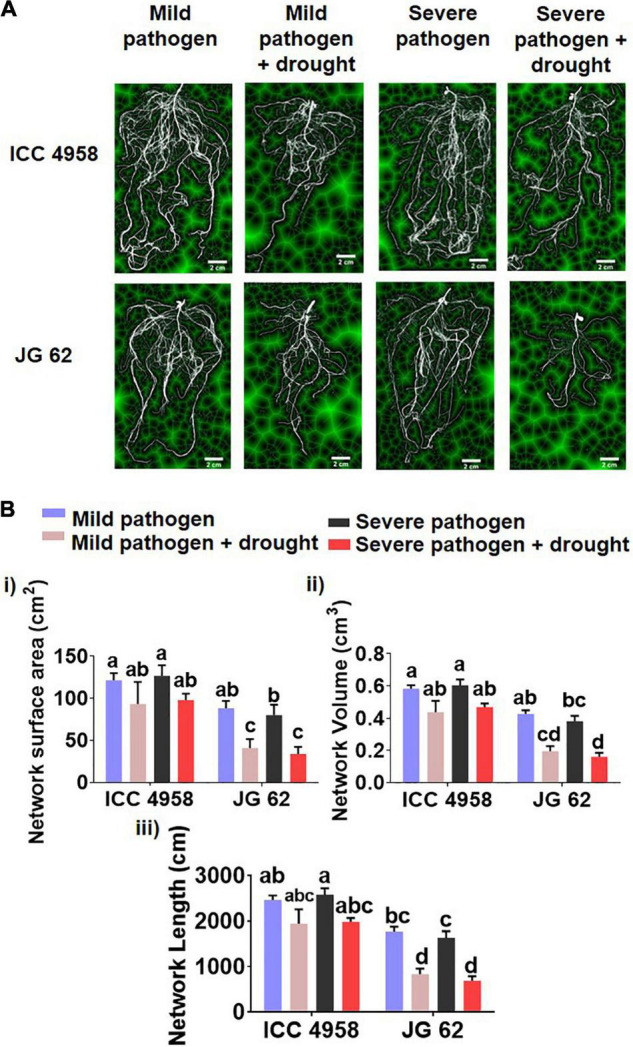
Differences in root architectural traits in the two chickpea genotypes across the different stress treatments**. (A)** A representative segmentation figure panel from the root scanning image analysis of ICC 4958 and JG 62 plants subjected to the mild pathogen, severe pathogen, mild pathogen + drought, and severe pathogen + drought. The analysis was done using Scanjet G4050 Photo Scanner and the images were analyzed using GIA roots (Galkovskyi et al., 2012). **(B)** The root trait variations were observed in the genotypes across the treatments. The image analysis data was accrued from the images of washed roots of 43 days old plants grown in greenhouse conditions using field soil from location 1. The bars on the graph indicate the averages of different traits for 3 RCB replicates with standard error as an error bar. Statistical significance difference between means is checked by one-way ANOVA and Tukey’s posthoc test. A significant difference between the mean at *p* < 0.05.

**FIGURE 3:**
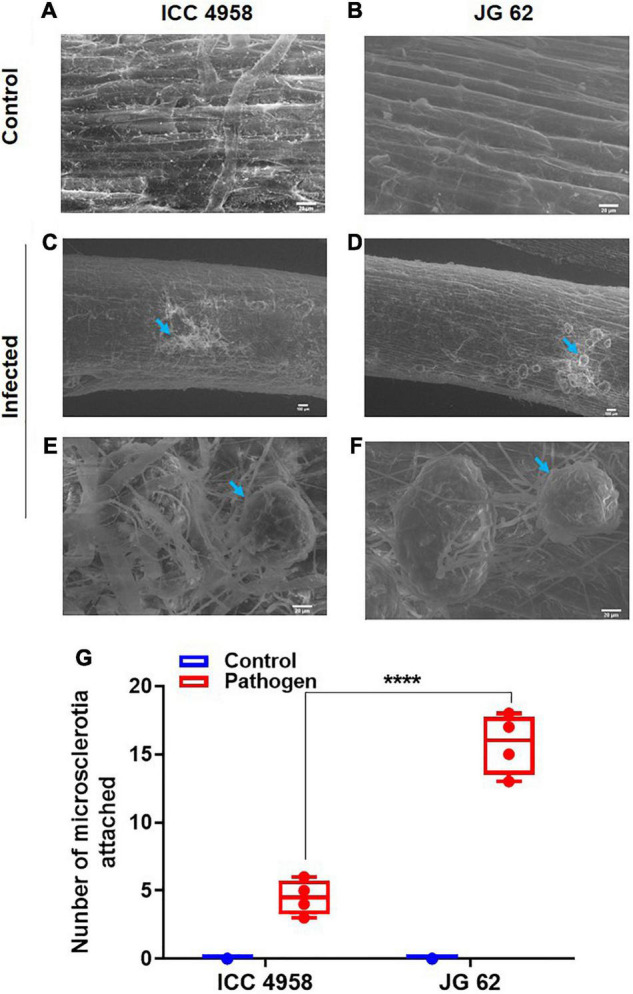
Root images indicating the differential attachment of *M. phaseolina* microsclerotia on the ICC 4958 and JG 62. ICC 4958 showed resistance to DRR pathogen (MH509971.1) infection. ICC 4958 and JG 62 were subjected to infection by microsclerotia under well-watered conditions using the blotting paper method (Irulappan and Senthil-Kumar, 2021). Roots were collected and examined for the number of attached microsclerotia using the scanning electron microscope. **(A)** Uninfected root images of ICC 4958. **(B)** Uninfected root images of JG 62. **(C)** Infected ICC 4958 roots showing attached microsclerotia. **(D)** JG 62 roots show the attached microsclerotia. **(E)** Mycelial growth on ICC 4958 roots. **(F)** Extensive growth of mycelia from the attached microsclerotia in JG 62. **(G)** The graph shows the variation in attached microsclerotia. Two-way ANOVA was used. Asterisks show the significance at ****p ≤ 0.0001. *N* = 5. The experiment was repeated three times at least.

To further explore the relationship between the ability to maintain water status and disease resistance, we compared the root growth characteristics of the two genotypes under individual and combined drought and pathogen stresses (Figure 2). The two genotypes grown under greenhouse conditions displayed significant differences in root growth associated traits *viz.*, root network area, root length, and volume (Figures 2A,B). ICC 4958 exhibited widespread and 1.4 fold longer roots with 1.36-fold larger network area, at 43 days after sowing (DAS) as compared to JG 62 under unstressed conditions. Under drought conditions, both the genotypes exhibited a substantial reduction in the root growth traits. However, the reductions were more significant in JG 62. Likewise, combined drought and pathogen infection reduced the root network area, volume, and length in both the genotypes, the reductions being more significant in JG 62 (Figure 2B). Although not very significant, similar reductions were made when we compared the root architectural traits of the two genotypes under different treatments at the podding stage (Supplementary Figure 14). To further explore the response of the two genotypes toward DRR infection, we examined plant roots infected by *M. phaseolina* for the number of attached microsclerotia. We observed that the number of microsclerotia attached over the roots of two genotypes varied significantly. The percentage of microsclerotial attachment was 4-folds lesser in ICC 4958 than in JG 62 (Figure 3).

### Heat Stress Alters Disease Dynamics in the Resistant Genotype Under Drought Stress

**FIGURE 4:**
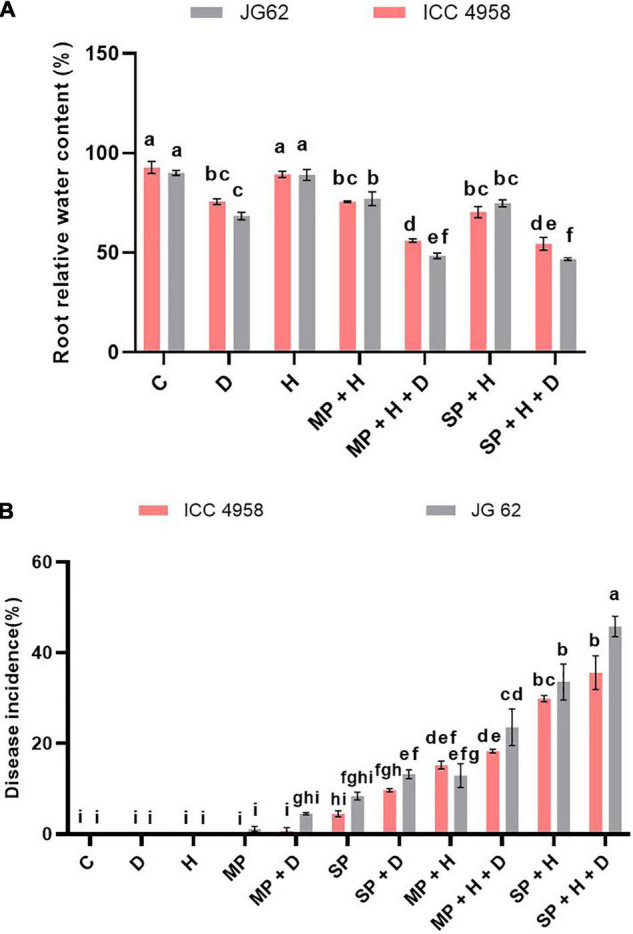
Combined drought and heat stress significantly alter the DRR disease development in chickpeas. Chickpea plants were exposed to different treatments namely, control, drought, heat, mild pathogen, mild pathogen + drought, severe pathogen, severe pathogen + drought, mild pathogen + heat, mild pathogen + heat + drought, severe pathogen + heat, and severe pathogen + heat + drought under field conditions during the off-season field trials. The control, drought, heat treatments were maintained separately in the isolated field and greenhouse conditions (Supplementary Figure 4). **(A)** Root relative water content of the two contrasting chickpea genotypes under different individual and combined stresses from the off-season field trials. **(B)** The DRR disease incidence in chickpea plants exposed to DRR under individual and combined drought and heat stresses. The treatment combinations are from both the on-season and off-season trials at Location 1. The bars in the following graphs are the average of respective block replicates with standard deviation as an error bar. Statistical significance between means was checked by two-way ANOVA and Tukey’s posthoc test.

**FIGURE 5:**
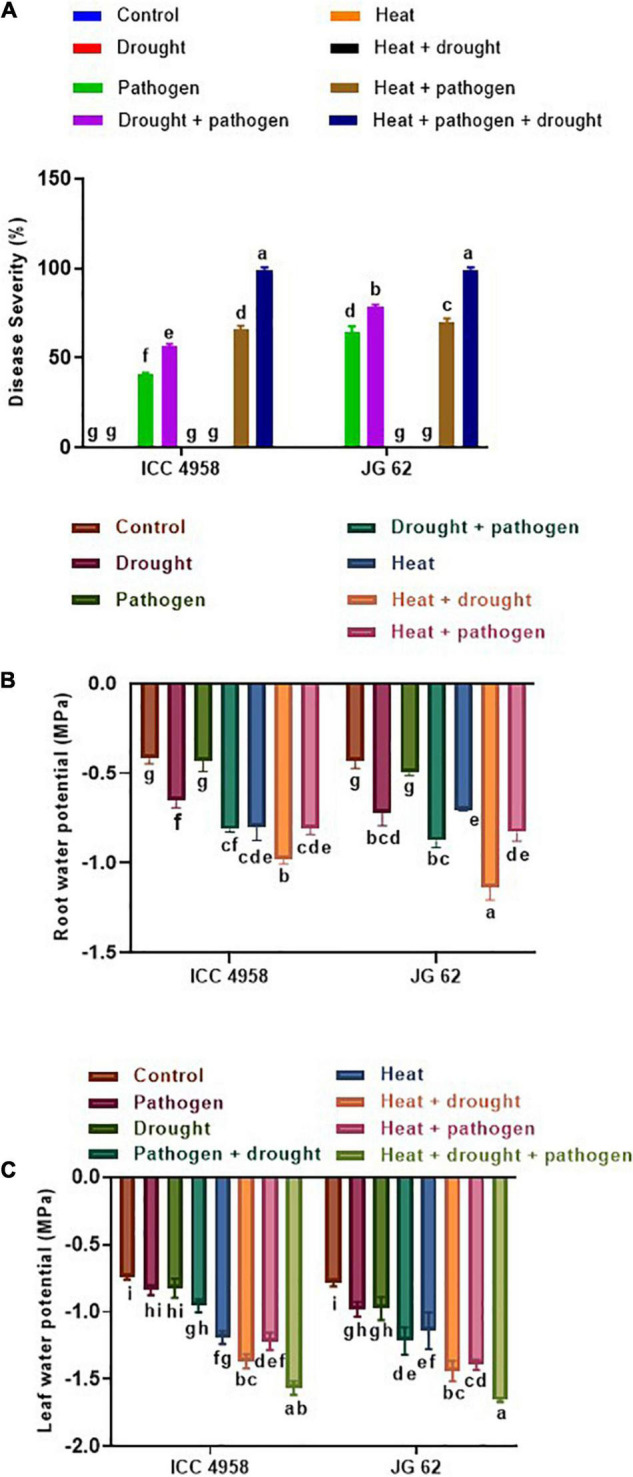
Effect of drought and heat stress on DRR disease severity in ICC 4958 and JG 62 chickpea genotypes. **(A)** Disease severity in the two genotypes under different stress treatments, namely control, drought, pathogen, drought + pathogen, heat, heat + drought, heat + pathogen, heat + drought + pathogen. **(B)** Root water potential **(C)** Leaf water potential of the individual and combined stressed ICC 4958 and JG 62 as measured on the 30th day post-drought imposition (Supplementary Figure 15). Root and leaf water potential were measured using Wiscor Psyprometer in 3 biological replicates per treatment. One replicate constituted three pots per set with each pot having two plants. Error bar signifies the SEM. Statistical significance difference between means is checked by one-way ANOVA and Tukey’s Posthoc test. The different letters denote a significant difference between mean at *p* < 0.05.

Plant water status is controlled by two major physiological processes, namely the absorption of water by roots and transpiration. The former is affected by the water level in soil, whereas the latter is influenced by the air temperature and relative humidity. Since we have found the possible relations between DRR incidence and root water status, we wanted to dissect the role of heat stress on DRR incidence. To achieve this, we included the off-season field trials characterized by increased daily temperatures, thereby including heat stress as an additional abiotic stress component. On comparing the on- and off-season field trial observations, we found that heat perhaps acts as another important abiotic factor modulating chickpea susceptibility to DRR under field conditions (Figure 4). We observed that root water status of both genotypes was significantly reduced under combined heat, drought, and pathogen stress (Figure 4A). We further observed that heat stress further escalated the disease incidence in both the genotypes (Figure 4B and Supplementary Figure 15). To further validate the involvement of heat in enhancing the susceptibility of chickpea to DRR, we evaluated the performance of ICC 4958 and JG 62 under the individual and combined heat, drought, and pathogen stress treatments (Supplementary Figure 16). Our results showed increased disease severity under combined stress treatments as compared to individual stresses (Figure 5A and Supplementary Figure 17). Disease severity increased significantly when DRR infection was accompanied by combined drought and heat stress treatments. Interestingly, the disease severity was higher under the combined heat and pathogen treatment than under the combined drought and pathogen infection in ICC 4958 (Figure 5A). We further compared the plant water relations of both the genotypes under the different stress treatments (Figures 5B,C). The maximum reduction in leaf water potential occurred under combined heat, drought, and pathogen treatment as indicated by significantly low leaf water potential under this treatment (Figure 5C). Root water potential could not be calculated for this treatment as we were unable to extract enough root sap required for the measurement. However, the measurement of the relative water content of roots under the triple stress treatment corroborated the observations of shoot water potential (Supplementary Figure 18). Here again, we found that combined heat and pathogen stress caused more significant reductions in root and leaf water potentials as compared to the combined drought and pathogen stress. We found a significant negative correlation between leaf water potential and DRR disease incidence (Table 1), suggesting that high plant water status might be one of the key parameters that could ward off DRR infection.

**TABLE 1:** Correlation analysis among disease susceptibility index, root, and leaf water potential.

	Root water potential	Leaf water potential	Disease susceptibility index
Root water potential	1		
Leaf water potential	0.881**	1	
Disease susceptibility index	0.034	−0.376	1

*Significance at p value of 0.05. **Significance at p value of 0.01. Difference between means was assessed by one way ANOVA and Tukey’s Post-hoc test.

### Influence of Edaphic Factors on Dry Root Rot Disease Incidence

To explore the effect of environmental conditions other than drought and heat in determining DRR incidence, the soil-nutrient analysis was performed across the experimental locations. Soil physicochemical properties such as sand and silt content, clay, pH, electrical conductivity (EC), and water holding capacity (WHC), along with the nutrient parameters like organic carbon (OC), phosphorous (P), potassium (K), nitrogen (N), sulfur (S), and boron (B) were assessed (Supplementary Table 5). DRR disease incidence under severe pathogen treatments observed across the three locations in on-season field trials was used for correlation analysis with the observed soil properties. Results revealed a positive correlation of DRR incidence with clay content and soil nitrogen availability. However, no significant correlation between DRR incidence and other soil properties could be found (Supplementary Table 6).

### Genetic Diversity Among Isolates of Macrophomina phaseolina

Pure isolates were obtained from culturing diseased root samples collected across different experimental and survey locations (Supplementary Figure 19). The fungus-specific regions were amplified using Internal transcribed space (ITS) based primers. The sequences were subjected to the phylogenetic analysis that grouped the isolates into three different major clades (I, II, and III) (Supplementary Figure 20 and Supplementary Table 7). The clades I and II could have originated from a common ancestor isolate. No cluster of isolates from a particular geographical region was seen. Incidentally, we also observed a wide variety of weeds in the sick plots, which were uninfected. The observations suggest a possibility of exploring the weeds for non-host resistance against *M. phaseolina* (Supplementary Figure 21 and Supplementary Table 8). However, further experiments in this regard are warranted.

## Discussion

### Root Water Relations Impact Dry Root Rot Disease Development

Extensive evidence on DRR incidence and its increased severity under deficit soil moisture conditions has been reported (Sharma and Pande, 2013; Sinha et al., 2019, 2021; Sharath Chandran et al., 2021). Our results further confirm that drought-induced plant water reductions may aggravate DRR infection in chickpea. Indeed, a significant correlation between the relative leaf water content and severity of charcoal rot was reported in common bean and soybean under water stress conditions (Yasmin et al., 2020), indicating the pathogen’s inclination for decreased plant-water content. In our study, a significant increment in DRR disease development was observed in chickpea plants exhibiting reduced root-relative water content in multiple field trial experiments. This indicates a high probability of DRR disease development under conditions that lead to reduced root water content. However, further experiments providing observations in a continuous-time interval could better establish the intricacies of the root-fungal interaction under drought stress. The positive association between soil moisture deficit and DRR disease development may be attributed to the inability of fungus to maintain sclerotia viability under higher soil moisture conditions and on the plant, tissues maintained at higher water levels (Marquez et al., 2021). *Macrophomina phaseolina* also exhibits the ability to have sustained growth and better survival under osmotic stress conditions due to the *de novo* synthesis of osmolytes such as glycerol (Baird et al., 2003) and variations in the lipid contents of both mycelium and microsclerotia (Marquez et al., 2021). On the plant side, weakened endodermal barrier and suppression of overall host defenses can cause enhanced DRR severity under the water stress conditions (Irulappan et al., 2022).

### Contrasting Genotypes Indicate the Role of Disrupted Root Water Relations in Enhancing Dry Root Rot

*Macrophomina phaseolina* is a root infecting pathogen and interferes with plant water relations. This fact supported by the observation that DRR infection is aggravated by drought compelled us to study the effect of DRR infection on two genotypes with contrasting drought tolerance. The ability of crops to absorb and transport nutrients and water is directly influenced by root system architecture (RSA), determined by the root length, root surface area, and root volume (Wang et al., 2019; Xiong et al., 2021), and root length density (Lynch et al., 2014; Zhan et al., 2015). ICC 4958 is known to have a robust root architecture with a dense root system (Kashiwagi et al., 2006). Earlier reports have shown that 35 days old ICC 4958 plants, sampled from two different seasons in the field and cylindrical experiments, had higher root length density than JG 62 (Kashiwagi et al., 2006). Since ICC 4958 also displayed enhanced DRR resistance, we were interested to see if the resistance could be related to the better root system architecture. As expected, we found that the root traits like root length, surface area, and volume varied significantly between the two genotypes across different treatments at the vegetative stage (Figure 2); ICC 4958 was found to possess a denser root system with a greater root area and volume as compared to JG 62. Since DRR symptoms are characteristically observed at later growth stages of chickpea, we observed the effect of pathogen infection on root architecture at podding stage under field conditions. Similar root trait differences between genotypes were found at podding stages but were found to be non-significant across the treatments (Supplementary Figure 14).

### Root Water Relations Play a Role to Enhance the Dry Root Rot Disease Development Under High Temperatures

Substantial yield losses in major crops (soybean, sorghum, and groundnut) due to *M. phaseolina* occur under high temperatures (Marquez et al., 2021). Likewise, high temperature predisposes chickpea to DRR (Sharma and Pande, 2013; Sharma et al., 2015). High temperature leads to enhanced proliferation of *M. phaseolina* in chickpea roots, as indicated by increased fungal DNA in infected roots of plants grown at 35°C as compared to those growing at ambient temperature (Sharath Chandran et al., 2021). Furthermore, elevated temperatures improved microsclerotia production (Marquez et al., 2021). Moreover, it is suggested that the heat stress might cause more damage to the root membrane than drought causing the enhanced release of root exudates into the rhizospheric soil (Chai and Schachtman, 2022). In the present study, the disease incidence observed under on-and off-season field trials showed an increasing trend under drought stress, and the effect was more pronounced under elevated temperature (Figures 4A,B). Higher root-relative water content reduced disease incidence under ambient temperatures but there was no clear trend under elevated temperatures (Figures 4A,B). We observed a significant difference in the DI of the two genotypes under the combined heat, drought, and pathogen stress (Figure 4B) indicating that the genotype JG62 was more infected under triple combined stress as compared to ICC 4958. The difference in the disease severity was not found to be statistically significant (Figure 5A). DI is measure of whether a plant is diseased or not. However, the disease severity reflects is the degree of disease symptoms. Results showed that the heat differentially break the resistance barrier in the contrasting genotypes (ICC 4958 and JG 62) and hence the variation in pathogen aggressiveness in colonization of the genotypes. Our results indicate that the resistance of ICC 4958 to DRR, exhibited under ambient temperature, was compromised under high-temperature conditions. This is in agreement with the concept that *M. phaseolina* plant mortality is driven by elevated soil temperature rather than moisture stress (Marquez et al., 2021). More precise controlled pot experiments further substantiated the effect of heat stress in altering plant water status and increasing disease severity (Figure 5). We also found a significant negative correlation between the leaf water potential and disease severity (Figure 5). A similar negative correlation was found between lesion length and leaf water potential in different sorghum genotypes subjected to *M. phaseolina* infection and post-flowering drought stress (Mayek-Pérez et al., 2002).

### Edaphic Factors Have a Mild Influence on Dry Root Rot Disease Incidence

The incidence of DRR disease is known to be impacted by several edaphic factors like soil pH, with the maximum DRR incidence being observed at pH 5.0 (Wagh, 2015). Our study indicated a positive correlation between clay content and DRR incidence (Supplementary Table 5). Clay content was reported to positively correlate with overall water retention through the increased specific surface area and occurrence of micropores (Reichert et al., 2009). Although the overall water retention increases under increased clay content, the amount of plant-available water is meager (O’Geen, 2012). Clay and sandy soil characteristics favor DRR occurrence (Wagh, 2015; Sinha et al., 2021). However, the amount of silt in the soil had a negative correlation (Sinha et al., 2021) with DRR incidence. We also show a positive correlation between soil nitrogen content and DRR incidence. Although the association between soil nitrogen content and DRR incidence has not been reported before, an increase in DRR incidence upon nitrogen fertilization is shown (Hemissi et al., 2018).

## Conclusion

**FIGURE 6:**
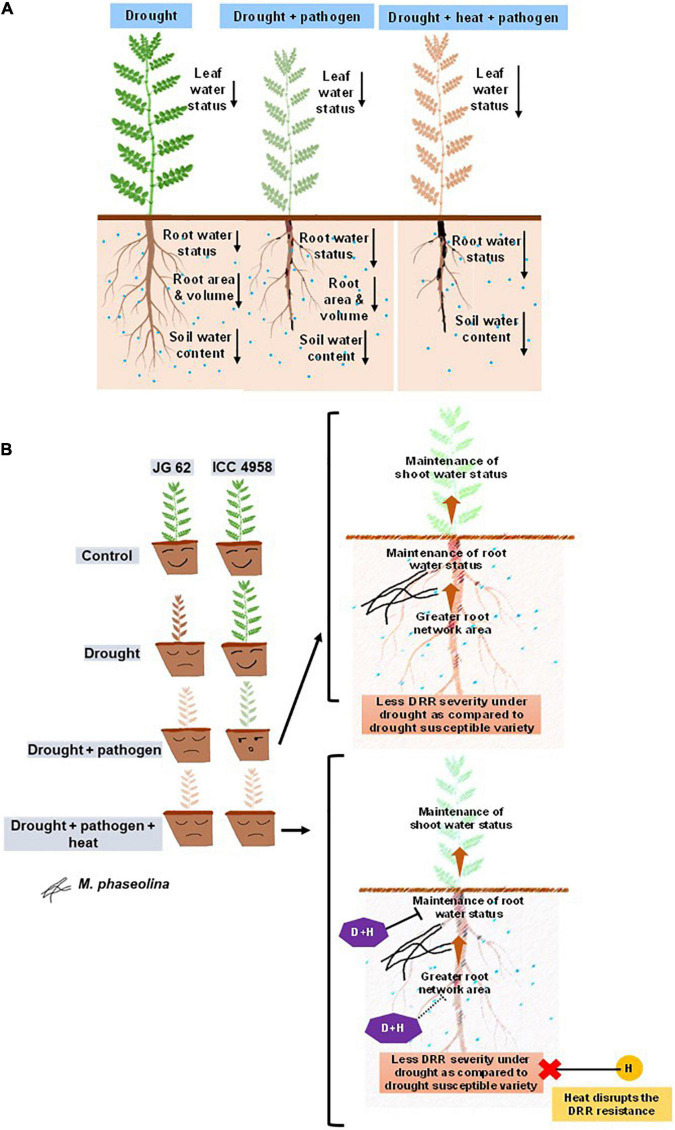
Schematic summary showing the effect of drought and heat on chickpea water relations and response to DRR. **(A)** Illustration comparing the effect of individual drought, drought + pathogen, drought + pathogen + heat on water relations of chickpea. Both drought-only and combined drought and pathogen treatments reduce the root and leaf water content apparently due to reduction in the root-associated traits like network area, volume, and length. Enhanced transpiration under heat stress further reduces the root water status. The differences in the leaf and root water status, root architectural traits, and soil water content in the three treatments are indicated by black-colored arrows. The length of the arrows represents the extent of reduction (the longer the arrows, the more are the reductions). **(B)** Schematic representation of the possible mechanisms by which heat can reduce the resistance of a drought-tolerant variety to DRR. The left-hand side panel indicates the differential response of a drought-tolerant and susceptible variety to drought, pathogen, and combination of drought, heat, and pathogen. Whereas ICC 4958 can resist DRR infection better under drought conditions, heat makes it significantly susceptible to DRR. The right-hand panel represents the possible mechanism behind the same. A combination of pathogen and heat stress additively reduces the plant water content and disrupts the resistance to DRR shown by ICC 4958 under drought stress conditions. We hypothesize that drastic reduction in root water content mediated by heat may be one of the primary physiological processes behind the loss of resistance of ICC 4958 under heat stress. Broken arrows exhibit a predicted observation warranting future investigations. P, pathogen stress; H, heat stress, D + H, combined drought and heat stress. In panel **(A)**, the colors of the shoots represent the effect of soil water deficit and pathogen infection on plants. Since *M. phaseolina* is a root infecting pathogen, symptoms are not very visible in shoots. A combination of drought and DRR pathogen leads to enhanced disease and reduced shoot water status indicated by the faded color of the shoots. The combination of heat, drought, and pathogen causes further additive reductions in leaf water status and an increase in disease severity which is indicated by the brown coloration of the shoot showing the maximum deleterious effect of the triple stress combination.

Our study demonstrated the impact of drought and heat stress in elevating the incidence of DRR in chickpea and enhancing plant mortality under both greenhouse and field conditions (Figure 6). The disease susceptibility could be related to plant water relations suggesting that drought-tolerant varieties with better physiological traits (deep, widespread, and turgid roots), and plant water relations can resist the DRR infection better. Furthermore, we discovered that heat stress, often associated with drought under field conditions, disrupts the DRR resistance in the drought-tolerant variety, suggesting a heat-induced breach of plant defense against the pathogen. Overall, we indicate the significant association of plant water status with DRR resistance and highlight the prospects to extensively investigate the influence of DRR infection on physiological and molecular processes involved in the maintenance of plant water status through mechanisms including root hydraulic conductivity. Considering the significant influence of drought and heat on chickpea resistance to DRR, we insist on the pertinence of screening for DRR resistance in chickpea genotypes under heat and drought stress conditions for identifying better breeding resources.

## Data Availability Statement

The original contributions presented in the study are included in the article/Supplementary Material, further inquiries can be directed to the corresponding author.

## Author Contributions

MS-K conceived the idea, planned the study, designed the experiments, and edited and finalized the manuscript. AC, KM, and VI executed the field and laboratory experiments and analyzed the data. AC, VI, BP, CP, VR, BM-R, and KR have contributed to the execution of field experiments at various locations. MK contributed to leaf and root water measurements. PP contributed to data analysis and organization. AC, PP, and MS-K drafted the manuscript. All authors approved the manuscript.

## Conflict of Interest

The authors declare that the research was conducted in the absence of any commercial or financial relationships that could be construed as a potential conflict of interest.

## Publisher’s Note

All claims expressed in this article are solely those of the authors and do not necessarily represent those of their affiliated organizations, or those of the publisher, the editors and the reviewers. Any product that may be evaluated in this article, or claim that may be made by its manufacturer, is not guaranteed or endorsed by the publisher.

## Footnotes

1
https://www.ibm.com/support/pages/downloading-ibm-spss-statistics-26

## Supplementary Material

Supplementary File 1 Raw data of all the figures and table presented in the manuscript.
