# Supplementary material for: Combined Drought and Heat Stress Influences the Root Water Relation and Determine the Dry Root Rot Disease Development Under Field Conditions: A Study Using Contrasting Chickpea Genotypes
Source: Front Plant Sci. 2022 May 9;13:890551. doi: 10.3389/fpls.2022.890551 (PMC9128860; doi:10.3389/fpls.2022.890551)
Supplement: Supplementary File 1 — Raw data of all the figures and table presented in the manuscript. [file Data_Sheet_1.zip › Supplementary Figures.PDF]

Supplementary Figures

Combined drought and heat stress influences the root water relation and determine the dry root rot disease development under field conditions: a study using contrasting chickpea genotypes

Authors:

Aswin Reddy Chilakala[1#], Komal Vitthalrao Mali[1#], Vadivelmurugan Irulappan[1], Basavanagouda S. Patil[2], Prachi Pandey[1], Krishnappa Rangappa[3], Venkategowda Ramegowda[4], M. Nagaraj Kumar[5], Puli Chandra Obul Reddy[6], Basavaiah Mohan-Raju[4] and Muthappa Senthil-Kumar[1*]

*Correspondence:
Muthappa Senthil-Kumar, National Institute of Plant Genome Research, Aruna Asaf Ali Marg, P.O. Box No. 10531, New Delhi 110067, India. Email: skmuthappa@nipgr.ac.in.

Supplementary Figure S1.

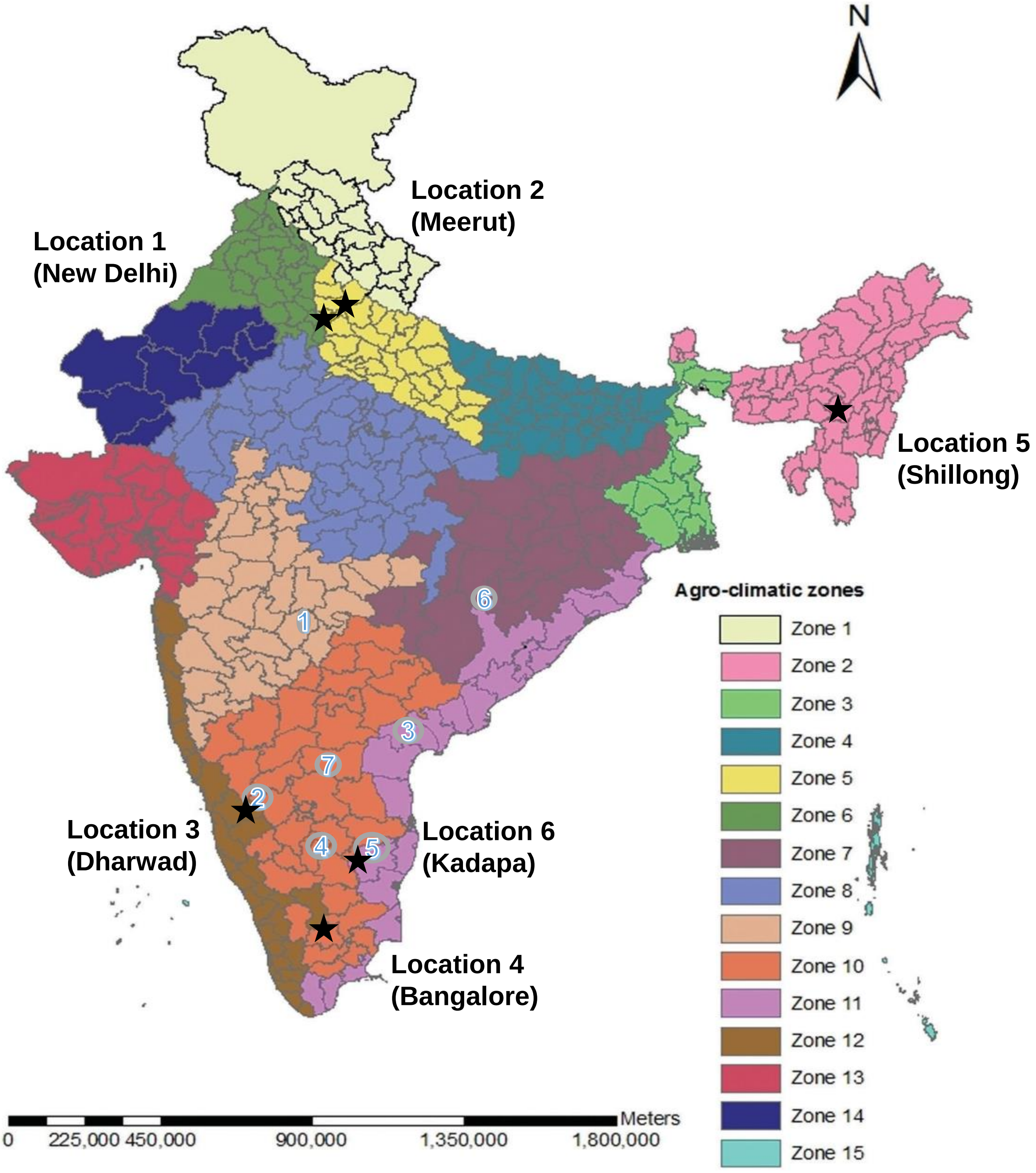

Supplementary Figure S1. **The different field trial and survey locations in the study with their agroclimatic zone distribution** (Agroclimatic map source: http://apps.iasri.res.in/agridata/19data/chapter1/db2019tb1_2.pdf). The current experimental locations and farmers' field surveys fall into eight major agro-climatic zones (zone 5, zone 6, zone 2, zone 7, zone 9, zone 11, zone 10, zone 12). The experimental locations are tagged in black letters whereas surveys locations that included villages of different districts (farmers' fields) were numbered in blue as 1- Akola (Maharashtra), 2- Dharwad (Karnataka), 3- Guntur (Andhra Pradesh), 4- Anantapur (Andhra Pradesh), 5- Kadapa (Andhra Pradesh), 6- Raipur (Chhattisgarh), and 7- Raichur (Karnataka) Zones were divided based on the Planning Commission (Khanna, 1989). Zonal names are listed below: Zone 1- Western Himalayan region, Zone 2- Eastern Himalayan region, Zone 3- Lower Gangetic plain region, Zone 4- Middle Gangetic plain region, Zone 5- Upper Gangetic plain region, Zone 6- Trans Gangetic plain region, Zone 7- Eastern plateau and hills region, Zone 8- Central plateau and hills region, Zone 9- Western plateau and hills region Zone 10- Southern plateau and hills region, zone 11- East coast plains and hills region, Zone 12- West coast plains and ghat region, Zone 13- Gujarat plains and hills region, Zone 14- Western dry region, Zone 15- Island region.

Supplementary Figure S2.

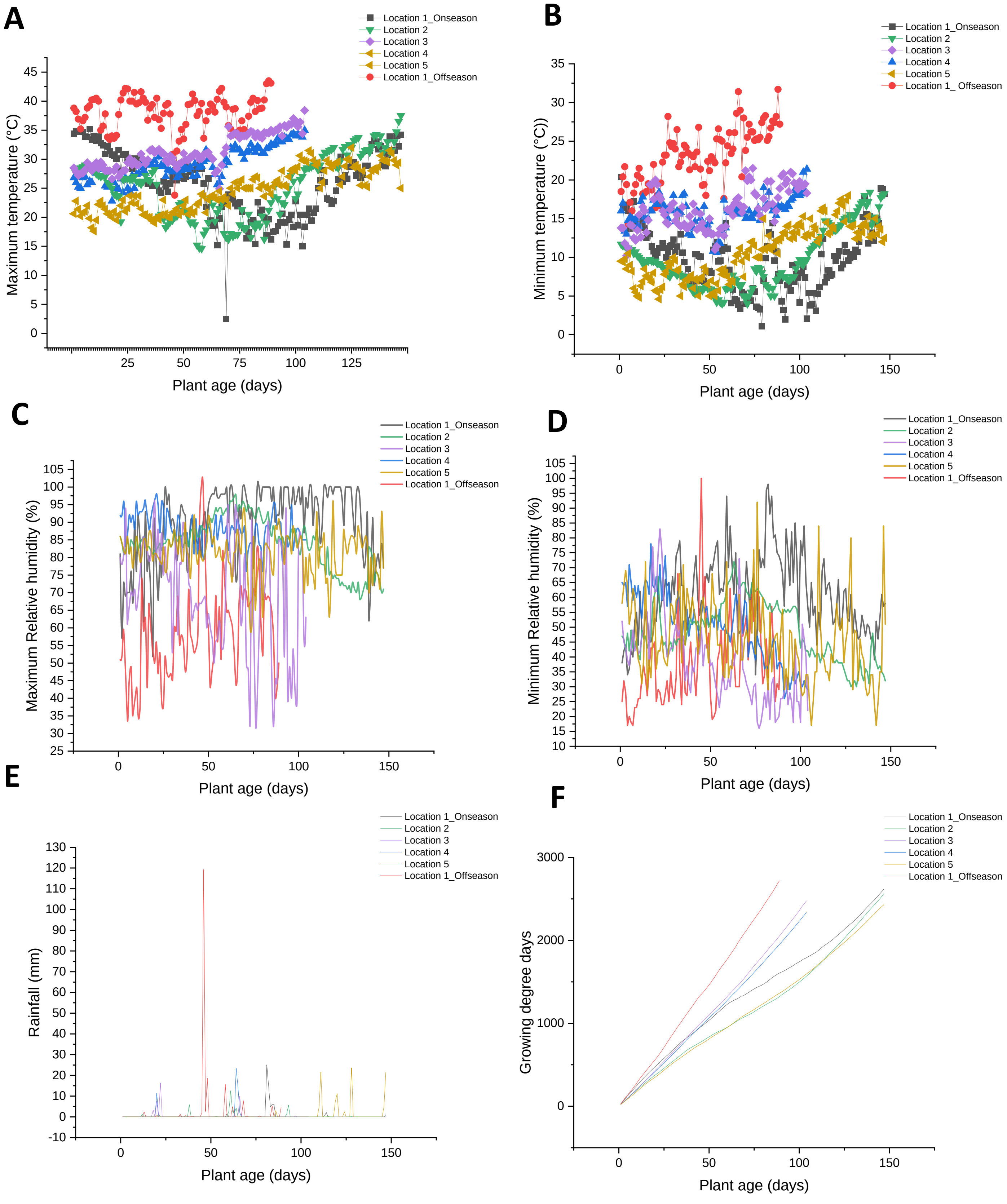

G.

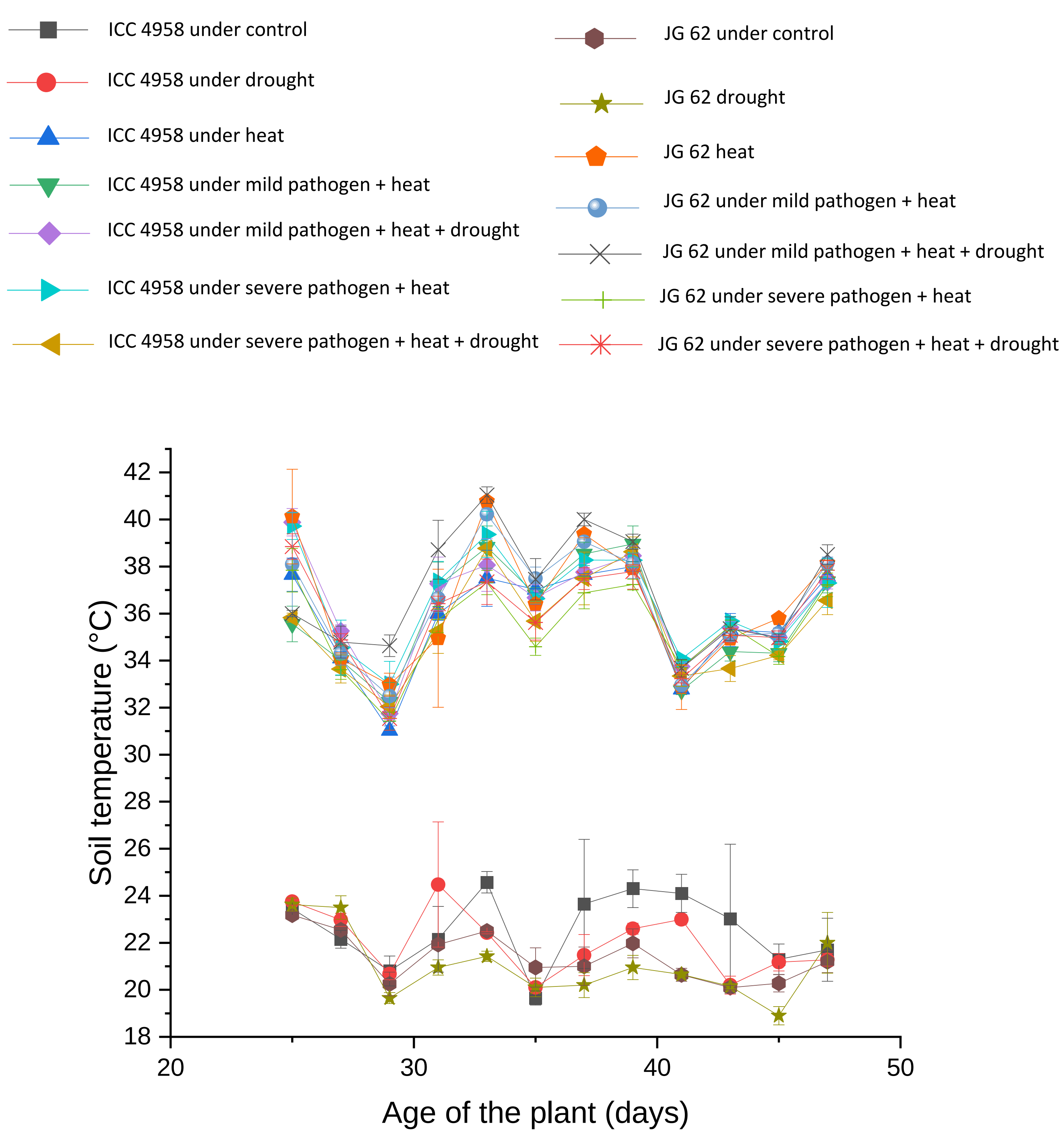

Supplementary Figure S2. Graphs indicating the weather conditions that prevailed during the experimental trials. (A) Graph plot indicating the daily maximum temperatures throughout the growth period of plants in all field trial locations. **(B)** Graph plot indicating the daily minimum temperature throughout the growth period of plants in all field trial locations. **(C)** Graph indicating the maximum relative humidity (%) throughout the growth period of plants in all field trial locations. **(D)** Graph indicating the minimum relative humidity (%) throughout the growth period of plants in all field trial locations. **(E)** Graph indicating the rainfall data during the crop growth in all field trial locations. **(F)** Graph indicating the daily accumulation of growing degree days during the crop growth in all field trial locations. (**G**) The soil temperature data of the off-season field trial conducted at Location 1**.** The soil temperature was measured in all the treatment plots on every alternate day 23 weeks. The measurements were made at the rhizosphere region (15-25 cm) from a minimum of four different points for every treatment plot. An average of four block replicates were plotted for the comparison. Error bars represent the standard error. Data of all the locations were collected at different regional weather stations located near them. The accumulated growing degree days (GDD) were calculated according to the formula: $GDD = Tmean - T_{base}$, if $Tmean > T_{base}$ and $GDD = 0$, if $Tmean < Tbase$. (https://mrcc.illinois.edu/gismaps/info/gddinfo.htm). Where the T_{base} is 0°C. Location 1- New Delhi –Regional Meteorological Center, New Delhi. Location 2- Meerut- Department of Soil Science, Sardar Vallabhbhai Patel University of Agriculture & Technology, Modi Puram, Meerut. Location 3- Dharwad- Main Agricultural Research Station, UAS Dharwad. Location 4- Bangalore –AICRP on Agrometeorology, GKVK, Bangalore. Location 5- Shillong- ICAR Research Complex for NEH Region, Umiam Research Station.

Supplementary Figure S3.

Block A		Block B		Block C		Block D
G2T2		G1T3		G1T1		G2T2
G1T2		G2T3		G2T1		G2T3
G1T3		G1T4		G1T3		G2T1
G1T4		G1T2		G2T4		G2T4
G1T1		G1T1		G2T3		G1T3
G2T3		G2T1		G2T2		G1T1
G2T4		G2T2		G1T2		G1T2
G2T1		G2T4		G1T4		G1T4

Treatments
T1=Mild pathogen
T2= Mild pathogen + drought
T3=Severe pathogen
T4=Severe pathogen + drought

Genotypes
G1= ICC 4958
G2= JG 62

Replicates
4 blocks

Supplementary Figure S3. The layout of treatment plots in randomized complete block design for the different field locations. Four blocks (A, B, C, D) were used for four replications. Each block contained all the four treatment (T1= Mild pathogen, frequent irrigation to maintain 80% field capacity (FC) + fungicide ; T2= Mild pathogen + drought, less frequent irrigation to maintain 50% FC + fungicide; T3= Severe pathogen, frequent irrigation to maintain 80% FC + no fungicide; T4= Severe pathogen + less frequent irrigation to maintain 50% FC + no fungicide) for both the genotypes ICC 4958 and JG 62. For control and drought treatment plot, seeds were treated with 10g/kg seeds each of Bavistin (active ingredient 50% WP Carbendazim, Hindustan Antibiotics Limited, Pune) and SAAF (active ingredient 12% WP Carbendazim plus 63% WP Mancozeb, United Phosphorus Limited, Mumbai) in 1:1 ratio. In the fields, Bavistin, and SAAF were added at the concentration of 2kg/ha. To manage soil and seed-borne disease infection in mild pathogen and mild pathogen with drought treatments, seeds were treated with a combination of Bavistin and SAAF @ 10g/kg of seeds. Also, soil drenching with a combination of fungicides (Mancozeb and SAAF in a concentration of 2 kg/ha and 1 kg/ha respectively) was followed on T1 and T2 to control the disease. The seeds sown in T3 and T4 plots were not treated with any fungicides.

Supplementary Figure S4.

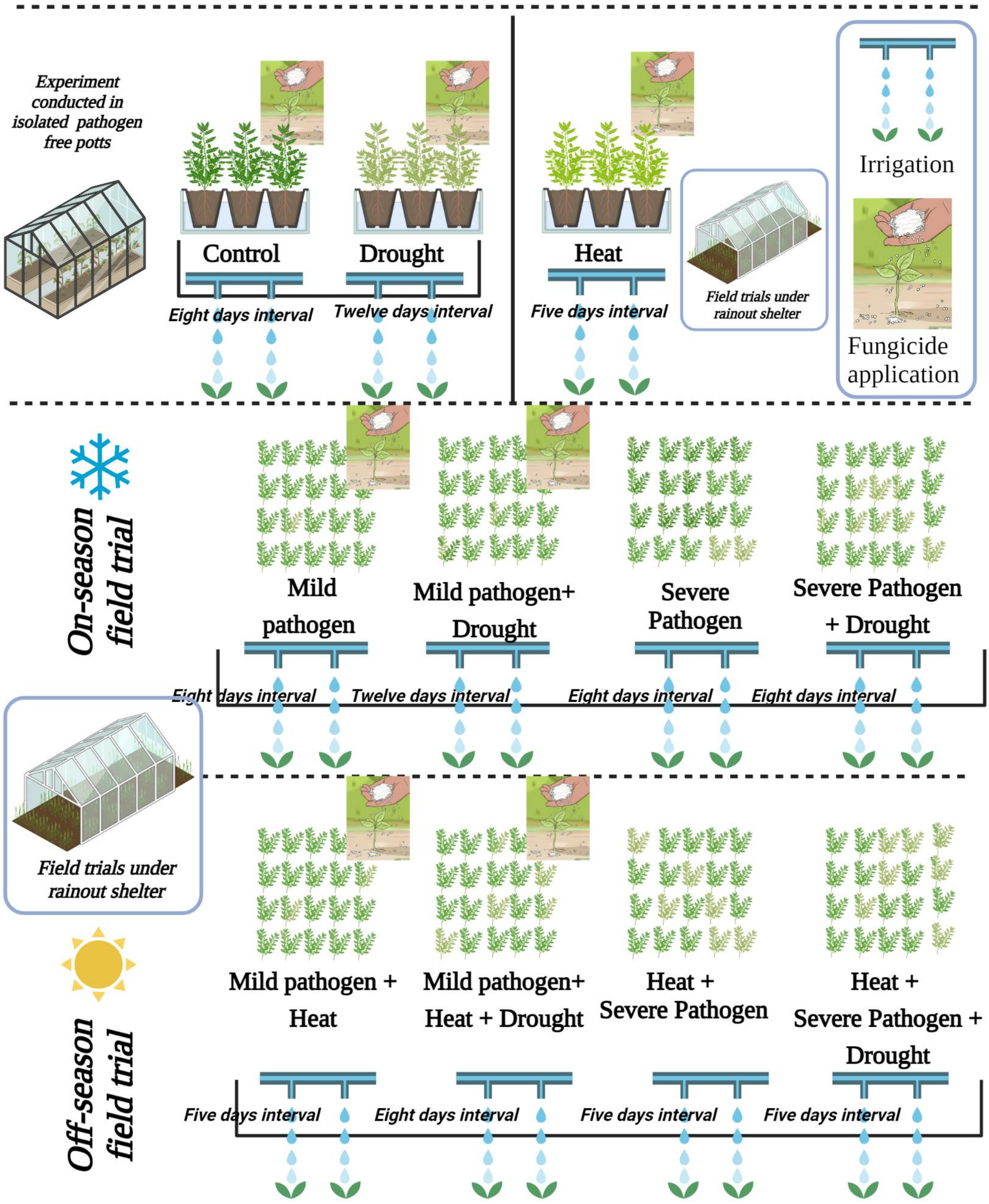

Supplementary Figure S4. Schematic representation of the treatment plans for the on- and off-season field trials. The control, drought, and heat stress treatment were provided in isolated pathogen-free plots. All treatments in the off-season field trials with drought stress as a component were conducted in rainout shelters. The control, drought, and heat treatments differed in irrigation schedule as indicated. During the off season field trials, the day temperature was considered as heat stress. The mild and severe pathogen with drought were irrigated whenever the soil moisture went below 50% FC, whereas, mild pathogen and severe pathogen stress plots were irrigated more frequently to maintain soil moisture of more than 80% of the field capacity. The soil moisture content was measured for all the treatments with a Lutron PMS-714 soil-moisture meter. The details of the different experiments and the parameters measured have been provided in Supplementary Table S4. The Figure was created using BioRender.com.

Supplementary Figure S5.

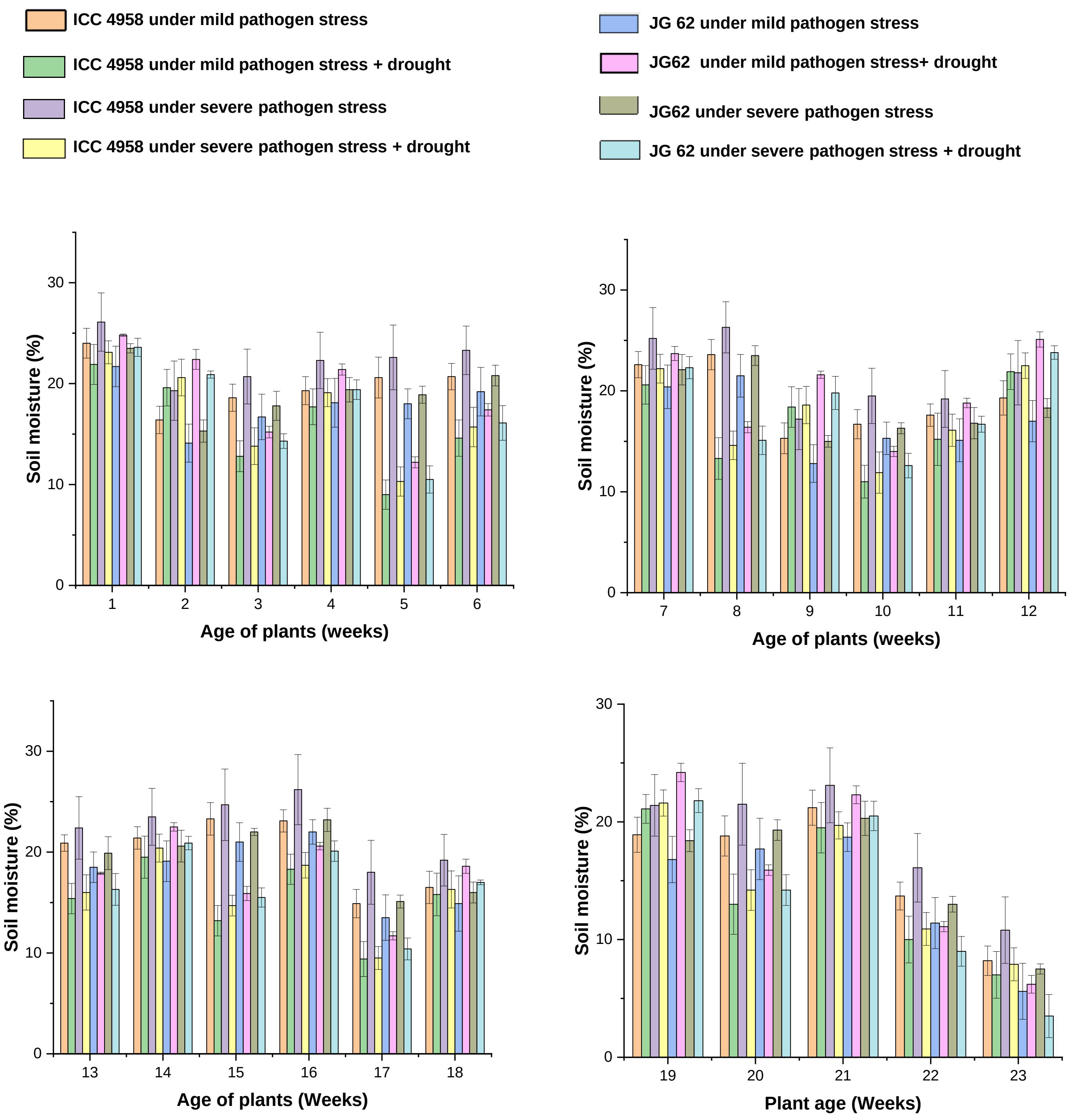

Supplementary Figure S5. The soil moisture data of the on-season field trial conducted at Location 1. The soil moisture content was measured in all the treatment plots on every alternate day using the Lutron PMS-714 soil moisture meter (Lutron Electronic Enterprise Co., Ltd., Taipei, Taiwan) for 23 weeks. The measurements were made at the rhizosphere region (15-25 cm) from a minimum of three different points for every treatment plot. An average of four block replicates were plotted for the comparison. Error bars represent the standard deviation.

Supplementary Figure S6.

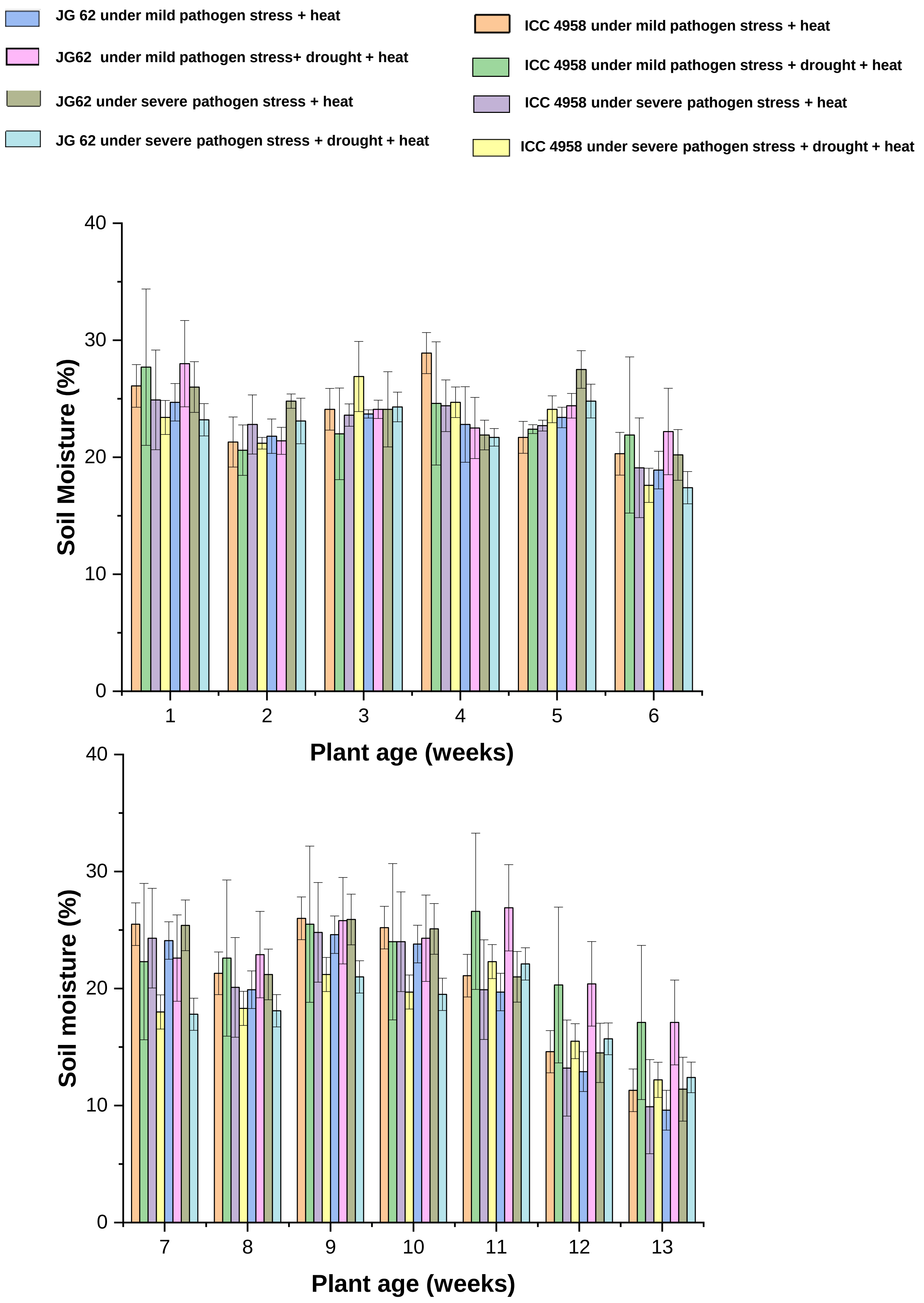

Supplementary Figure S6. The soil moisture data pertaining to offseason field trials. At field location-1, it was measured in all treatment plots on every alternate day using Lutron PMS-714 soil moisture meter (Lutron Electronic Enterprise Co., Ltd., Taipei, Taiwan). The measurements were made at the rhizosphere region (15-25 cm) at a minimum of three different points for every treatment plot. An average of four block replicates were plotted for the comparison. Standard deviation was given as an error bar for each data bar.

Supplementary Figure S7.

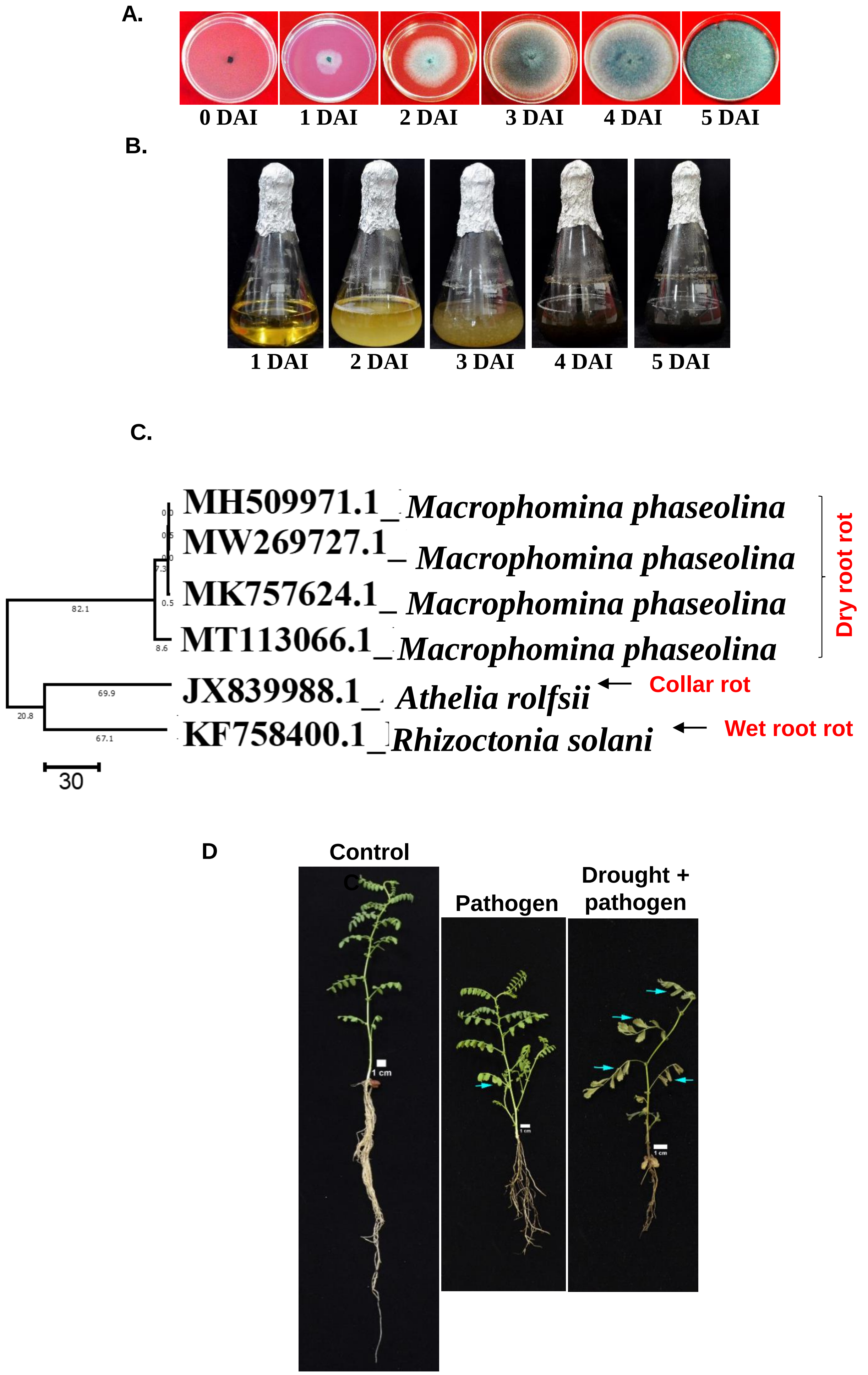

Supplementary Figure S7. Confirmation of pathogen isolated from fields. A small part of surface-sterilized fungal-infected root tissue taken from plant samples from Location 3 was cultured on PDA media. **(A)** Stages of fungal growth on PDA. **(B)** Stages of fungal growth on PD broth. The number at the bottom of the pictures in (A) and (B) represents the age of the culture. Further, genomic DNA was isolated from the fungal plate using DNAzol® Reagent, and ITS sequence was amplified by using universal ITS primers (ITS1-5' TCCGTAGGTGAACCTGCGG 3' and ITS4- 5' TCCTCCGCTTATTGATATGC 3'). **(C)** Phylogenetic tree showing the similarity between *M. phaseolina, Rhizoctonia solani* (causal agent of wet root rot, WRR), and *Athelia rolfsii* (causal agent of collar rot). The tree was constructed using UPGMA (MEGA7). The numbers indicate the branch length. **(D)** Chickpea genotype JG 62 was subjected to individual and combined drought and *M. phaseolina* infection. After the drought imposition, plants were uprooted and examined. Chickpea plants grown under well-watered conditions show healthy shoot and root. Plants treated with pathogen under well-watered conditions (90% field capacity) show symptoms with infected primary and lateral roots. Plants treated with combined drought (35% field capacity) and pathogen treatment show an infected plant with dry leaves (arrow mark), no lateral roots, and brittle primary roots.

Related references:

1. Sneath P.H.A. and Sokal R.R. (1973). Numerical Taxonomy. Freeman, San Francisco.
2. Felsenstein J. (1985). Confidence limits on phylogenies: An approach using the bootstrap. Evolution 39:783-791.
3. Nei M. and Kumar S. (2000). Molecular Evolution and Phylogenetics. Oxford University Press, New York.
4. Kumar S., Stecher G., and Tamura K. (2016). MEGA7: Molecular Evolutionary Genetics Analysis version 7.0 for bigger datasets. Molecular Biology and Evolution 33:1870-1874.

Supplementary Figure S8.

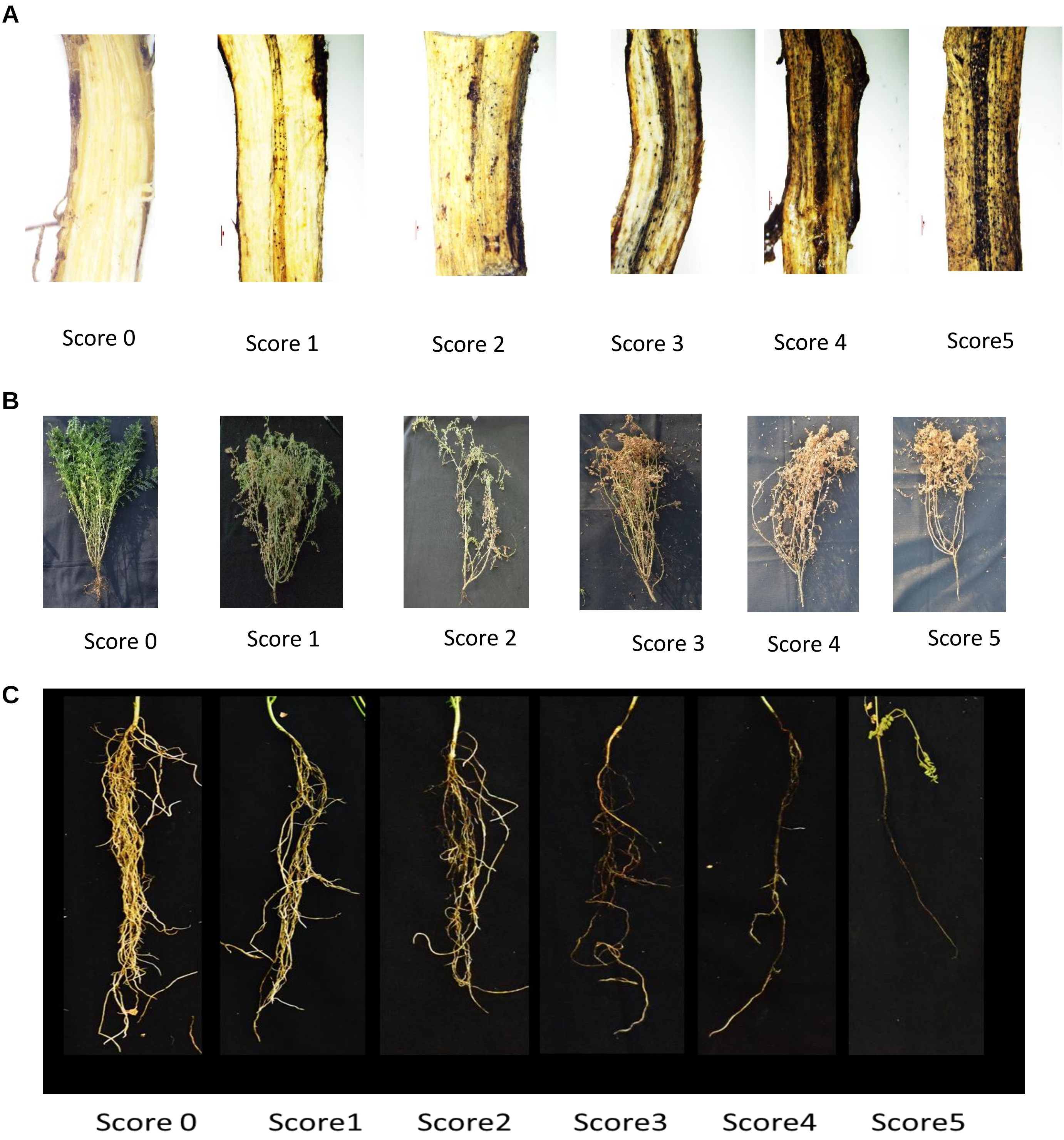

Supplementary Figure S8. Representative images for disease score based on disease severity. (A) Magnified split-open root images. **(B)** Individual plant images represent the disease severity based on foliar and root symptoms. **(C)** Root images from the infected plants grown in pots under greenhouse conditions. Score 0 indicates no disease, Score 1 to 5 represent a gradual increase in disease severity.

Supplementary Figure S9.

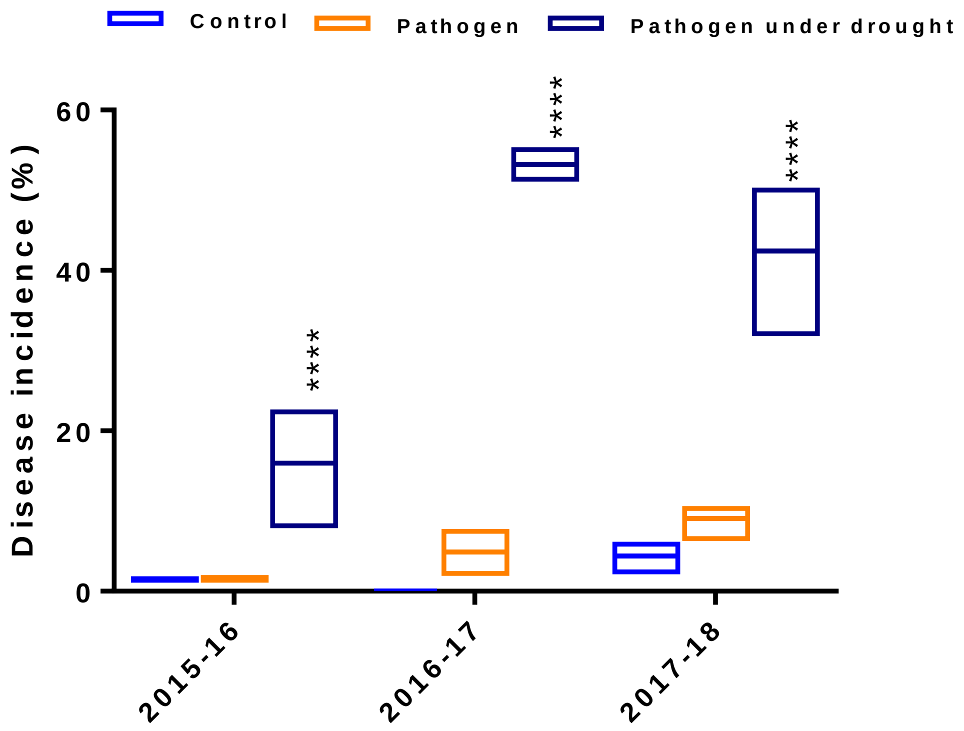

Supplementary Figure S9. Dry root rot disease incidence across years. Chickpea plants subjected to pathogen infection and drought stress exhibit different levels of disease incidence. The drought stress aggravated the incidence across the years (Sinha et al., 2019). Prism was used to analyze data. Data were analyzed using two-way ANOVA and Tukey's test to determine significance. Asterisk was used for indicating the significance levels.

Sinha, R., Irulappan, V., Mohan-Raju, B., Suganthi, A., & Senthil-Kumar, M. (2019). Impact of drought stress on simultaneously occurring pathogen infection in field-grown chickpea. *Scientific reports*, 9(1), 1-15.

Supplementary Figure S10.

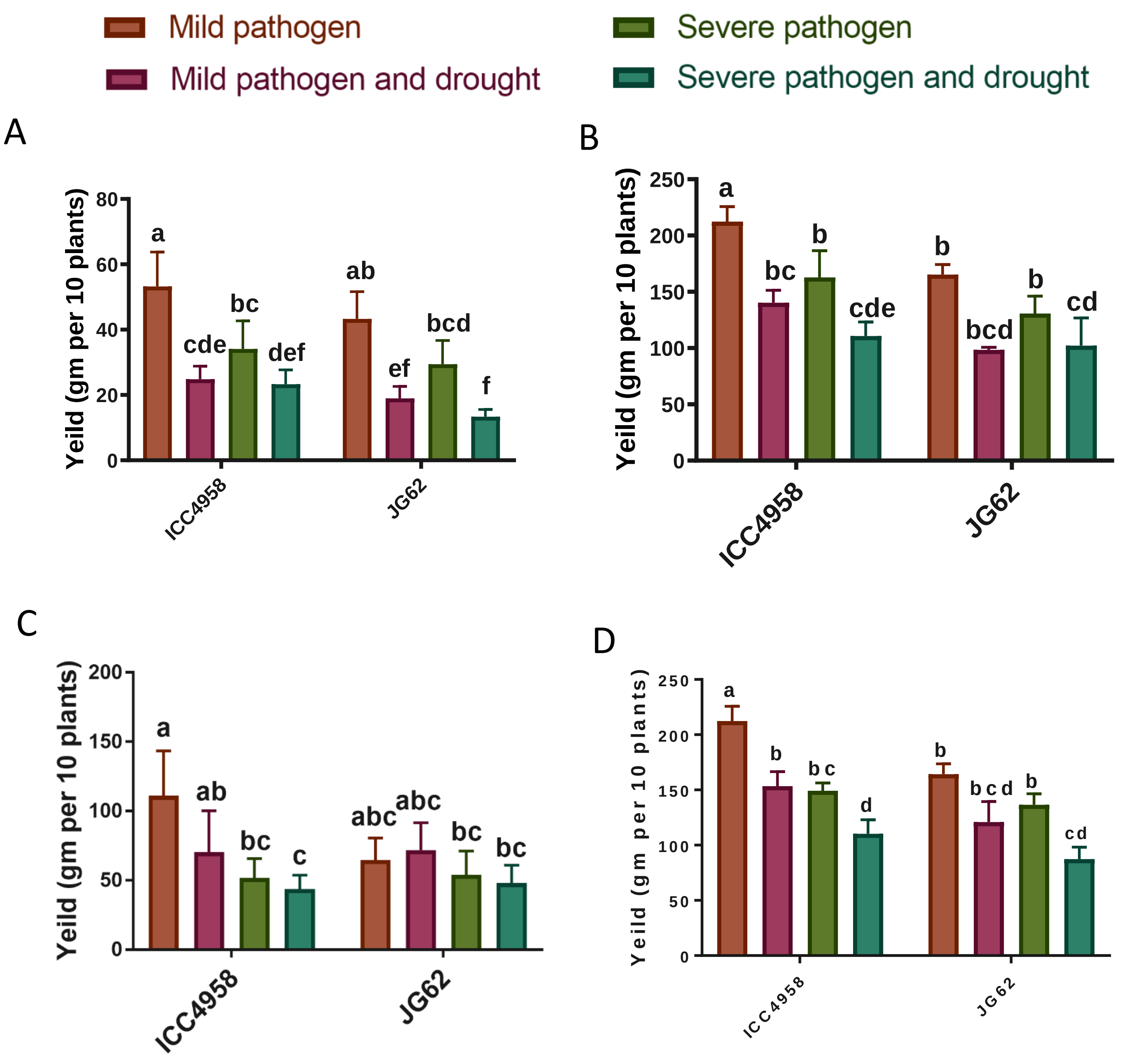

Supplementary Figure S10. Total pod yield of chickpea plants sown in four locations. Total yield was calculated for field trial year 2020-21 for **(A)** Locations 1 **(B)** Location 2, **(C)** Location 3, **(D)** Location 4 (details of the locations are given in Supplementary Table S1). Each bar for yield in the graph is the average of three RCB replicates with SEM indicated by the error bar. Statistical significance difference between means is checked by one-way ANOVA and Tukey's Posthoc test. The different letters denote a significant difference between the mean at $p<0.05$.

Supplementary Figure S11.

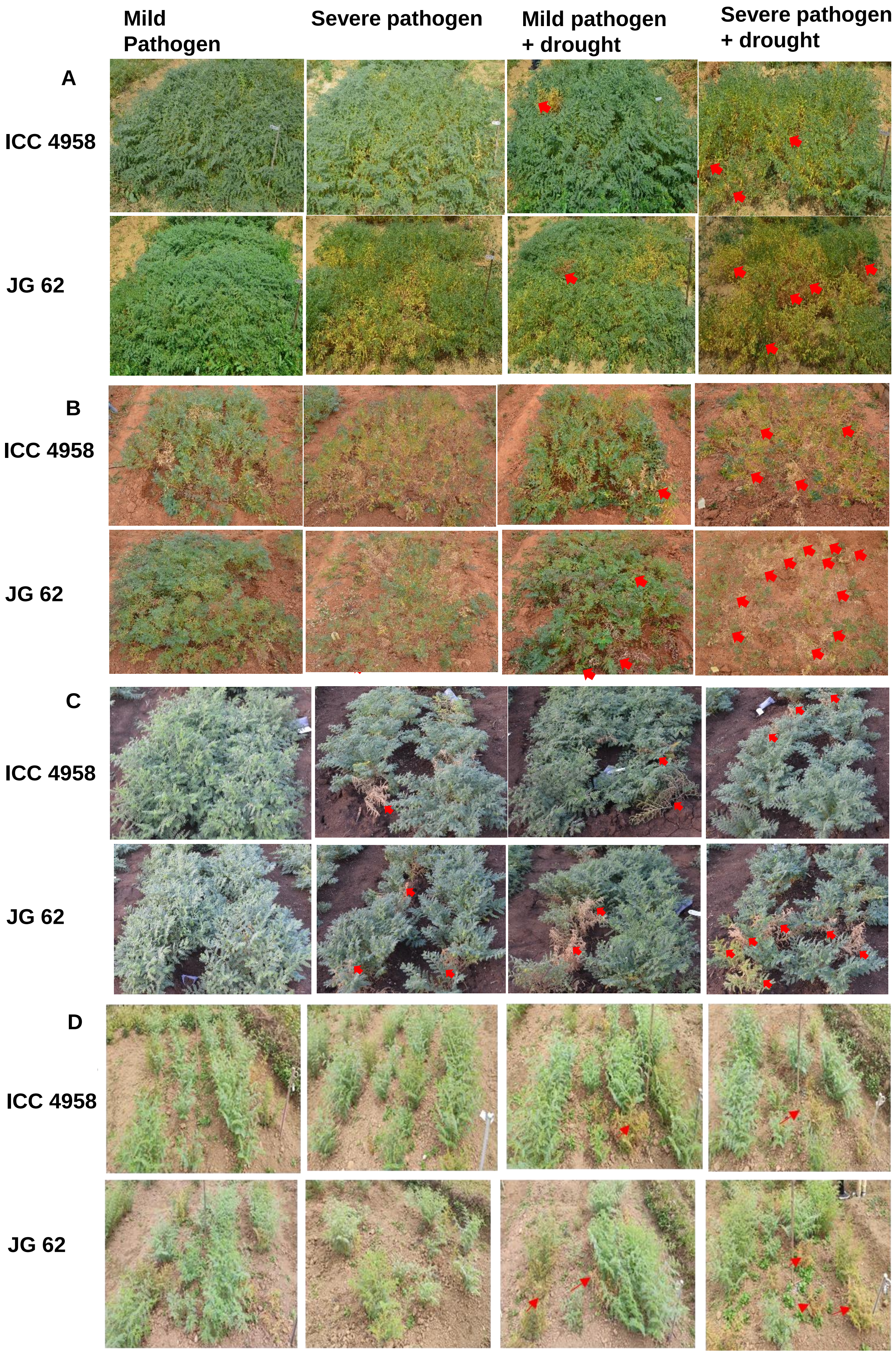

Supplementary Figure S11. Field view of different locations showing the DRR disease in the fields. (A) Representative images of plants at the podding stage from location 2. **(B)** Field image showing chickpea plants infected with DRR at a vegetative stage at location 4. **(C)** Field image showing chickpea plants infected with DRR at a vegetative stage at location 3. **(D)** Field image showing chickpea plants infected with DRR at a vegetative stage at location 5. All four treatments viz. mild pathogen, mild pathogen + drought, severe pathogen, severe pathogen + drought are indicated. Red-colored arrows point at the infected plants.

Supplementary Figure S12.

A.

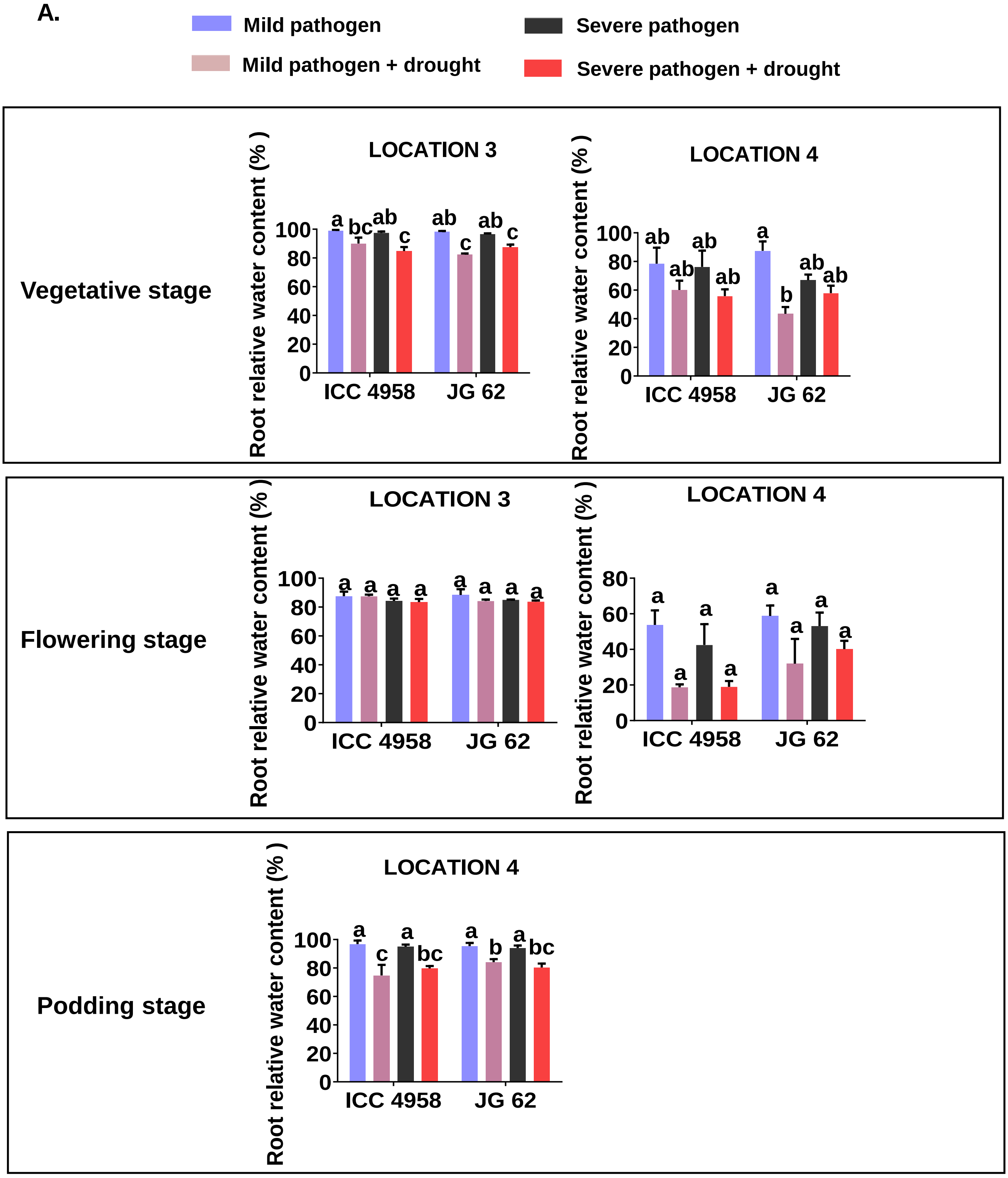

B.

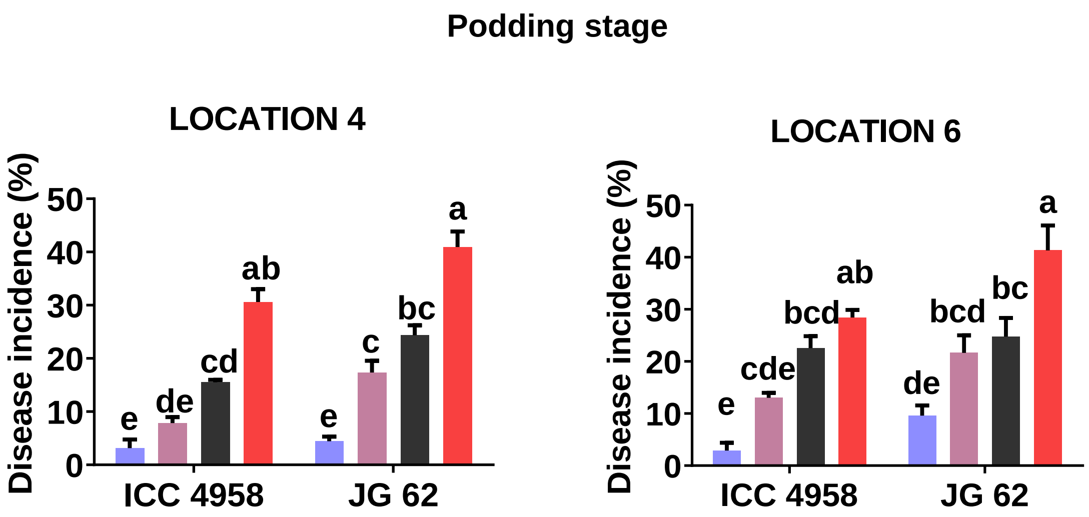

Supplementary Figure S12. Plant water status and disease incidence of chickpea genotypes under different stress and their combinations across different experimental locations. **(A)** Root relative water content of ICC 4958 and JG 62 at vegetative and flowering stages for different locations. **(B)** Disease incidence was observed across the experimental locations in the on-season field trial. The respective stages at the time of measurements are indicated. The bars in the graph are the average of respective block replicates. Error bars indicate standard deviation. The bars on the graph indicate the averages of different treatments for 4 replicates with standard error as an error bar. Statistical significance difference between means is checked by two-way ANOVA and sidak's mean multiple comparison test. The ** denotes $p<0.01$, **** denotes $p<0.0001$, and ns denotes non-significant. Location 1 – New Delhi (at the podding stage), Location 2- Meerut, Location 3- Dharwad (Vegetative, Flowering and podding stages), Location 4- Bangalore (at the podding stage), Statistical significance between means were checked by two way ANOVA and Tukey's Posthoc test. The different letters denote a significant difference between mean at $p<0.05$.

Supplementary Figure S13.

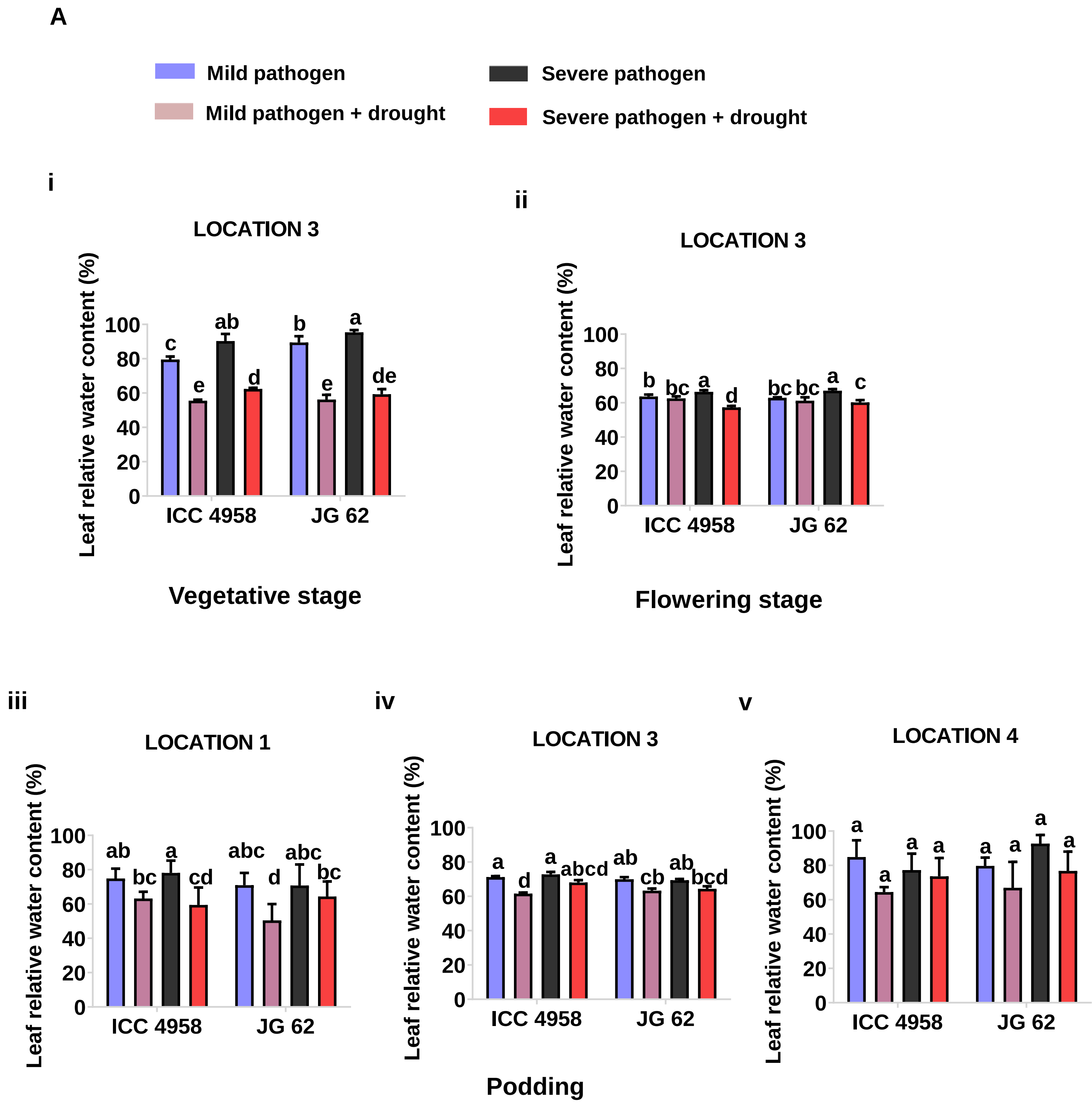

B

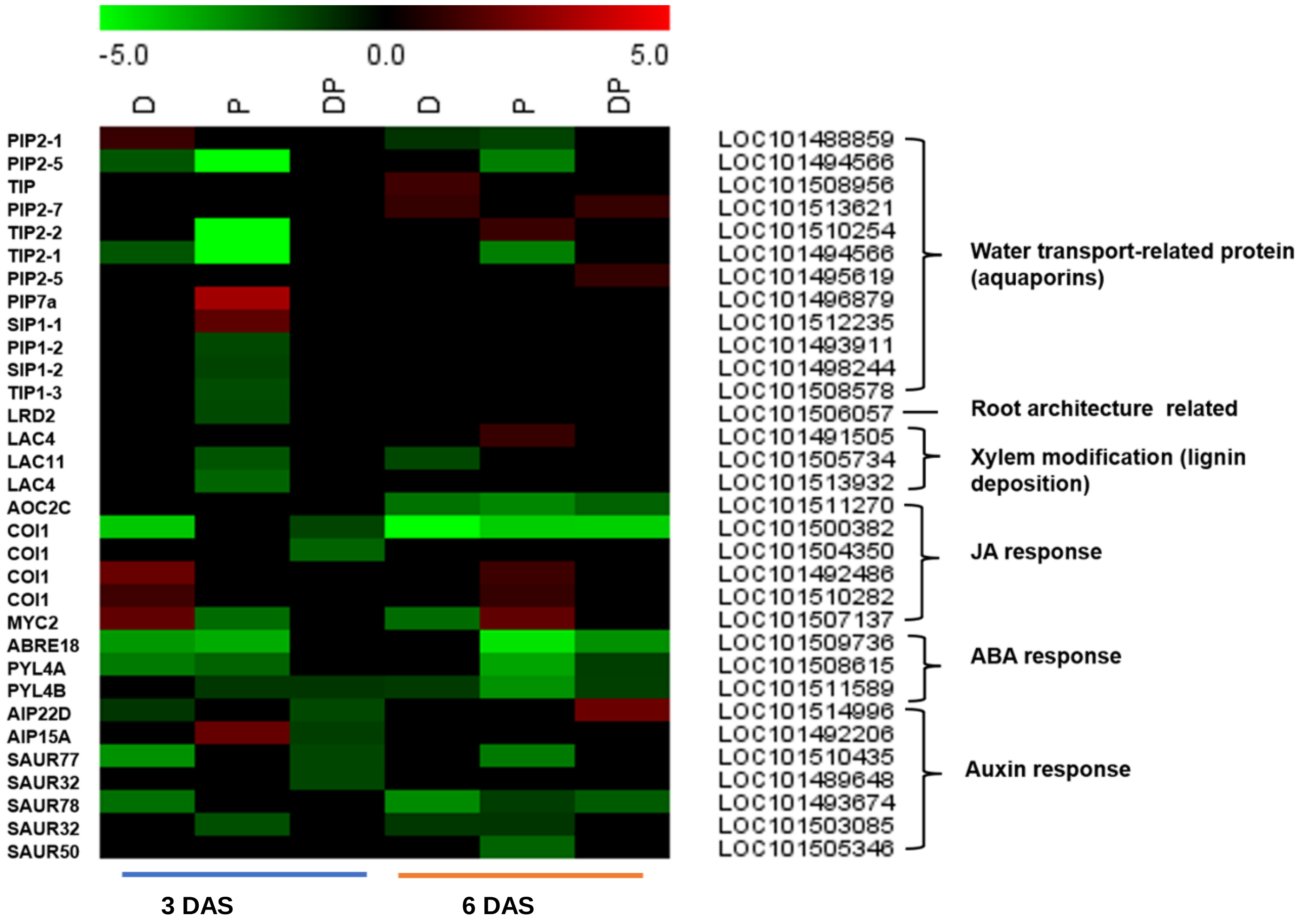

Supplementary Figure S13. Effect of drought, pathogen stress and their combination on plant water status and expression of related genes in different chickpea genotypes. (A) Relative water content in leaves of chickpea genotypes under different stress and their combinations across different experimental locations. Leaf relative water contents of the two genotypes at the different locations. The RWC measurements were made at different growth stages like vegetative (i), flowering (ii), and podding (iii-v). The respective stages at the time of measurements are indicated. The bars in the graph are the average of respective block replicates. Error bars indicate standard deviation. Location 1 – New Delhi (at the podding stage), Location 2- Meerut, Location 3- Dharwad (Vegetative, Flowering and podding stages), Location 4- Bangalore (at the podding stage), Statistical significance between means were checked by two way ANOVA and Tukey's Posthoc test. The different letters denote a significant difference between mean at $p<0.05$. **(B) Heat map showing the change in gene expression** at 3 and 6 DAS in JG 62 under different stress treatments. Heat map shows the fold change in expression of differentially expressed genes involved in water transport, root architecture, xylem modification (lignin deposition) and hormone response (auxin, ABA, JA) under drought (D), pathogen (P), drought and pathogen (DP) stress treatment as compared to control (Irullappan et al., 2022). DAS- days after sowing.

Supplementary Figure S14.

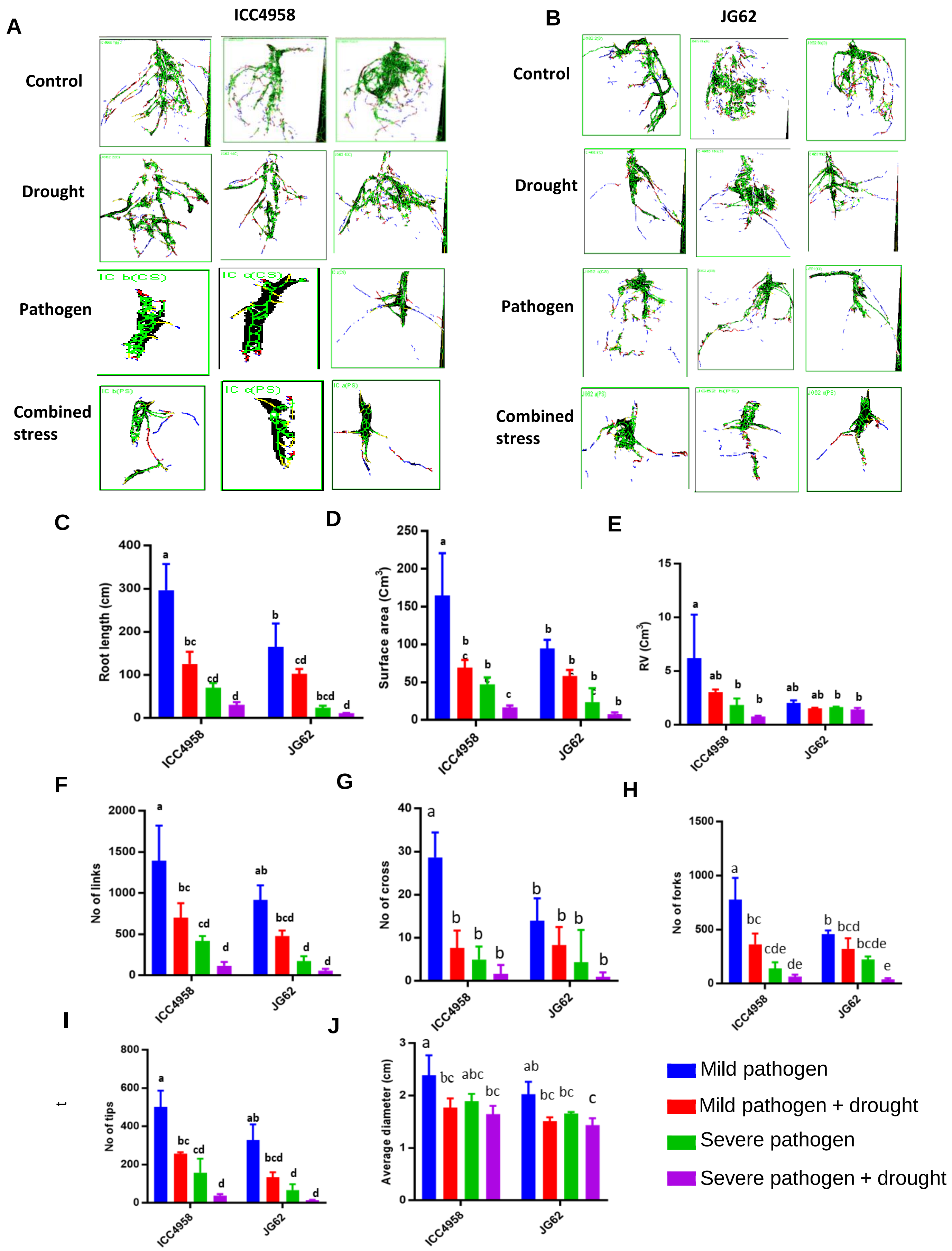

Supplementary Figure S14. Root architecture of ICC 4958 and JG 62 under drought and combined stress. (A) Scanned images of ICC 4958 roots under the different treatments **(B)** Scanned images of JG 62 roots under the different treatments showing the changes in root traits. The images were captured and processed from all the treatment plots using the WinRHIZO root scanners (Reagent instruments, Quebec, Canada) for studying the details of root morphology and two-dimensional architectural parameters. Bar graphs indicating the changes in root traits like **(C)** root length, **(D)** surface area, **(E)** root volume, **(F)** number of links, **(G)** cross, **(H)** forks**,** and **(I)** tips along with **(J)** the average diameter of roots. Root sampling was done at the podding stage following the protocol described by Bohm (Bohm, 2012) from Location 6. Representative roots from each treatment plot sampled across all the treatments were used in the analysis. Each bar in graphs is the average of 3 RCB replicates with SEM as an error bar. Statistical significance difference between means is checked by one-way ANOVA and Tukey's Posthoc test. The different letters denote a significant difference between mean at $p<0.05$. Root architecture was analyzed by root system imaging using WinRHIZO professional software for recording the details of root morphology and two-dimensional architectural parameters like total root length, root surface area, root volume, number tips, and several links for all four treatments.

Supplementary Figure S15.

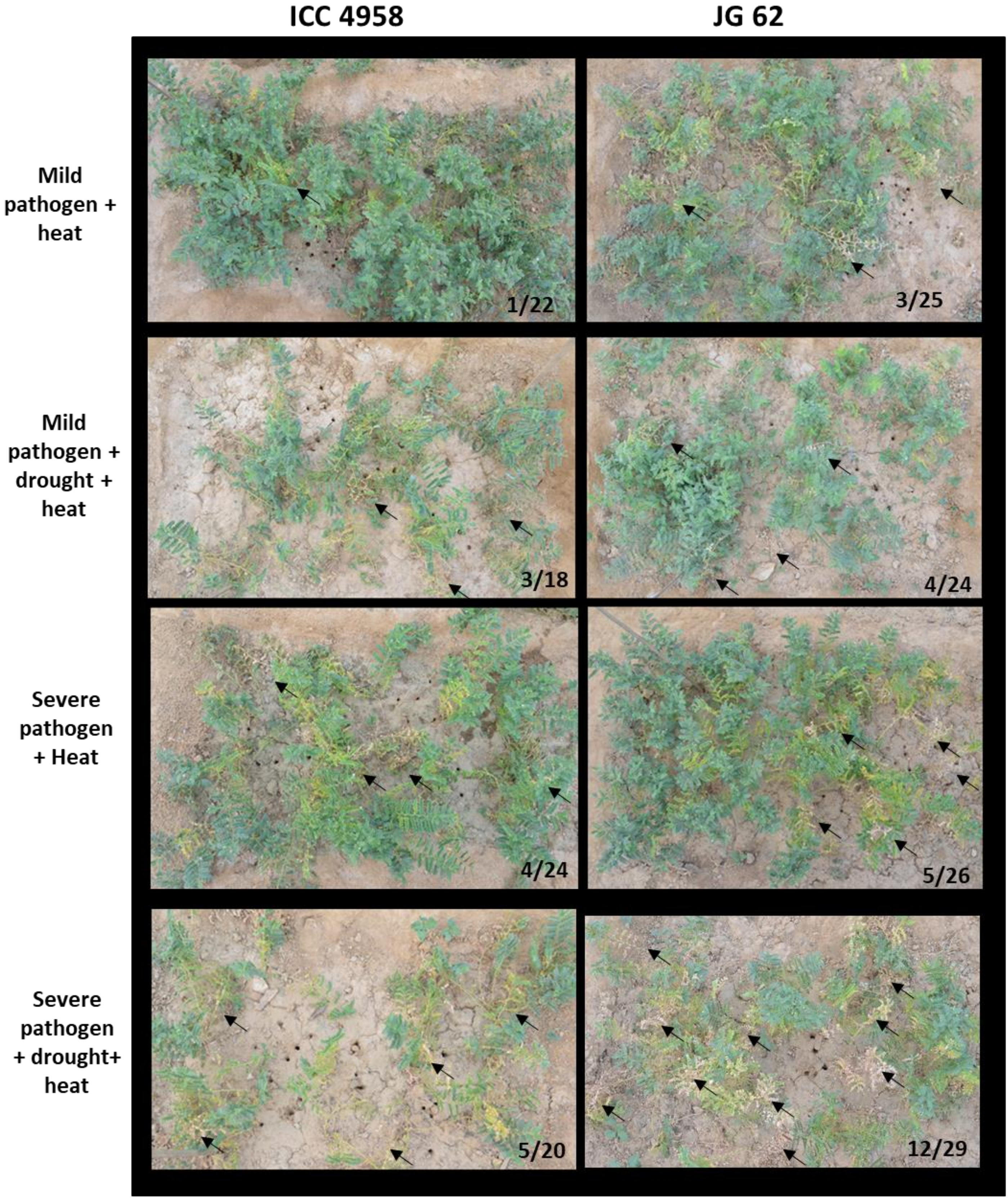

Supplementary Figure S15. Effect of combined drought, heat, and pathogen on chickpea plants. The representative images of experimental plots. Black color arrows point at the diseased plants. The numbers at the bottom indicate the number of infected plants/ total number of plants.

Supplementary Figure S16.

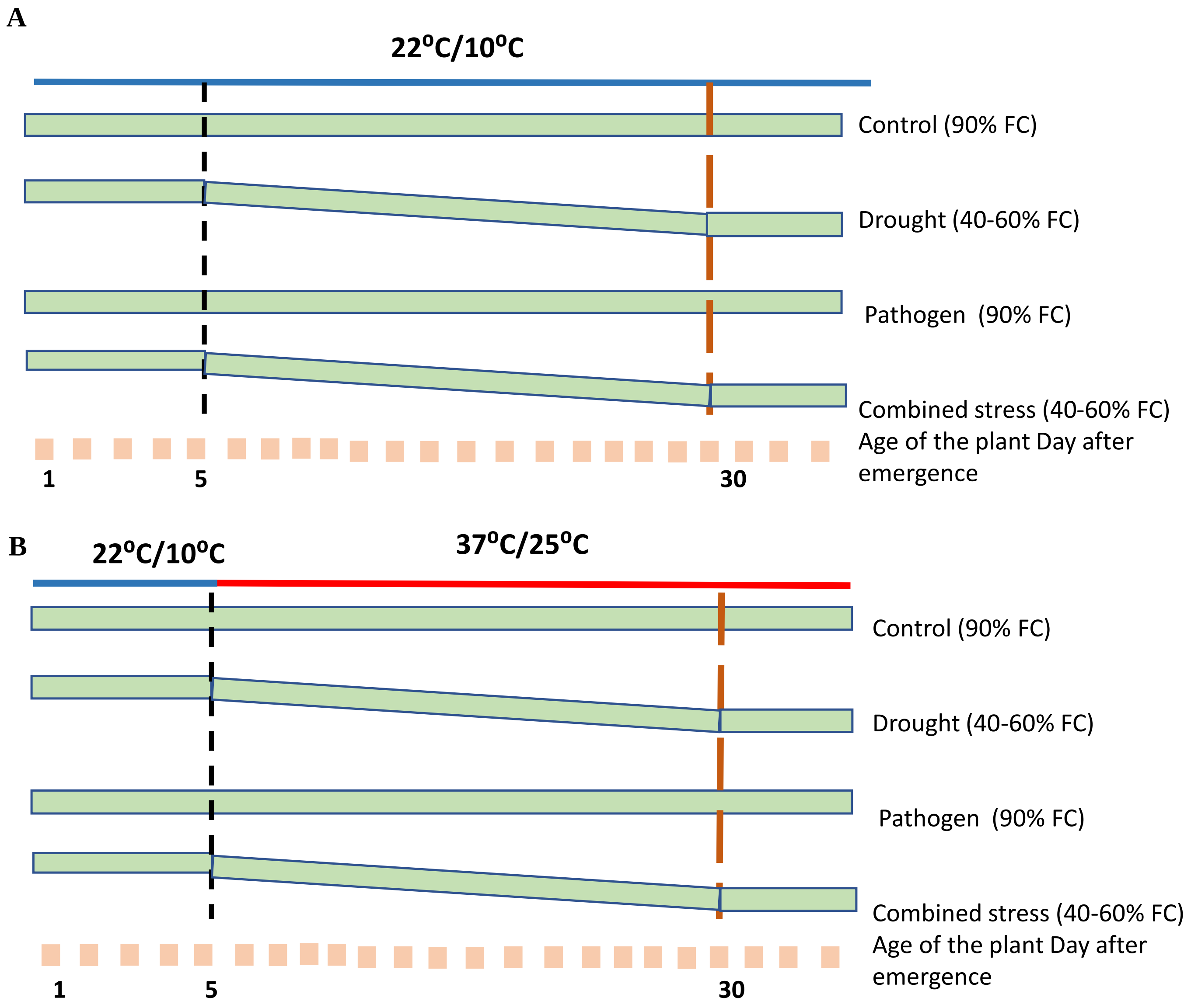

Supplementary Figure S16. Outline of the pot experiment with triple combined stress in chickpea. (A) A pot experiment with three treatments namely, pathogen only, drought only, and combined drought and pathogen along with control was performed to study the effect of drought stress on DRR disease progression in chickpea genotype ICC 4958 and JG 62. The experiment was conducted using ten pots (at least 10-20 biological replicates) for each treatment. Surface sterilized chickpea seeds were used in the experiment. For pathogen and combined stress treatment, plants were grown in a sick pot containing *Macrophomina phaseolina* inoculum whereas, control and drought treatment were grown in autoclaved soil rite. All plants were maintained at 90% field capacity (FC) for the first five days. Desired drought level (40-60%FC) in drought and combined stress treatment was achieved in 16 days at 22/10ºC (optimum temperature) by following the gravimetric method. Control and pathogen treatments were maintained at 90% FC. **(B)** Protocol for the imposition of triple combined stress. The protocol was similar to (A) except that heat stress treatment (37/25ºC) was imposed 5 days after sowing in combination with drought. The drought was maintained for the next five days by replenishing the water loss due to evapotranspiration. Samples were collected for relative water content measurement and microscopic observations on 15 and 30 days post combined stress. The orange-colored dotted line indicates the age of the plant after germination and black and brown-colored dotted lines indicate the days post combined stress treatment. The black vertical line indicates the start of drought imposition and the brown vertical line indicates the start of combined stress treatment. The solid line indicates the field capacity level of treatments and the dotted line indicates the days of the experiment. The blue and red solid line indicates the temperature treatment for a particular time. In heat, drought, and pathogen stress treatment, the temperature at the earlier stage from emergence to the 5th day was 22 ºC/10º till the exposure to heat stress when the temperature was raised to 37/25ºC.

Supplementary Figure S17.

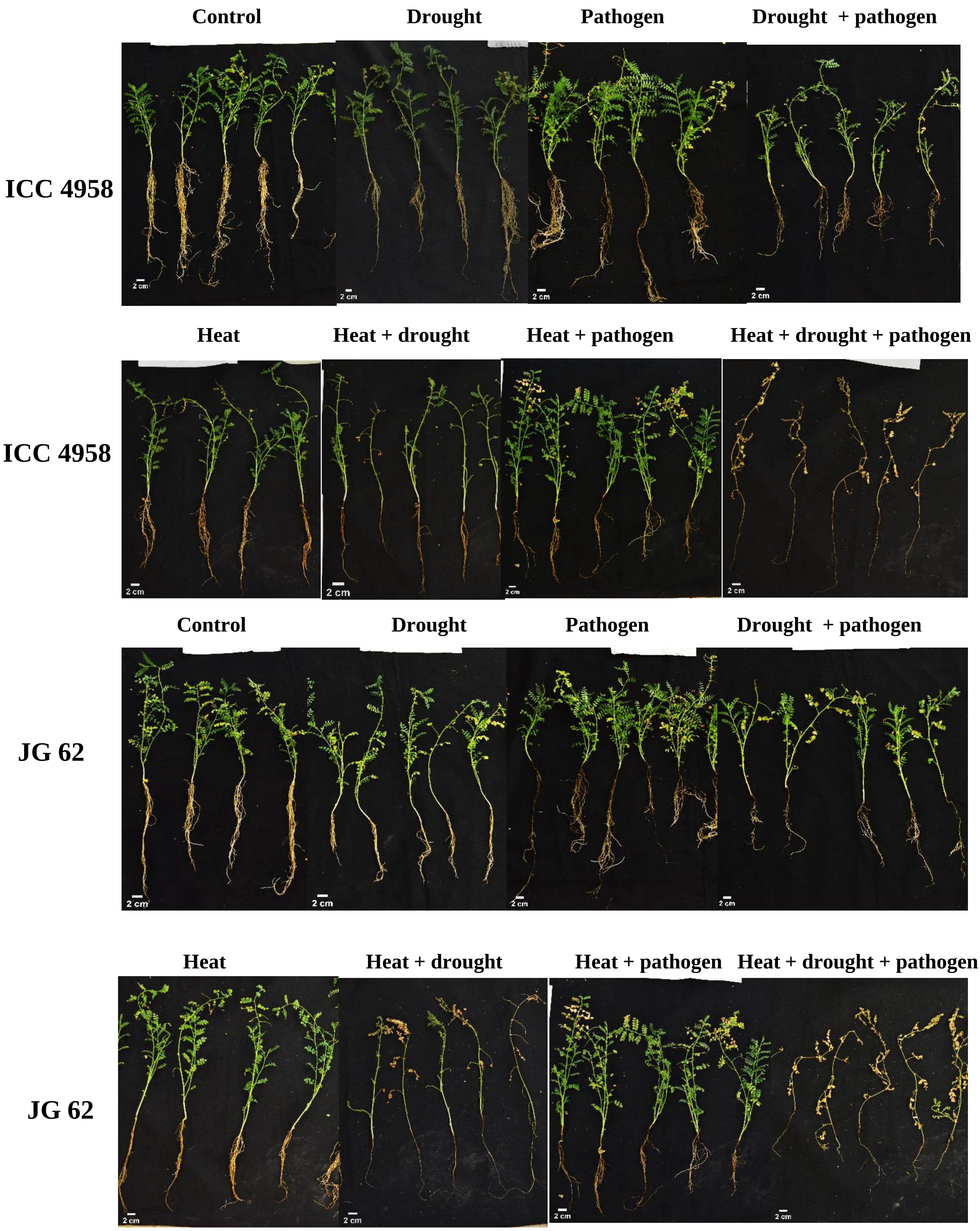

Supplementary Figure S17. Effect of drought and heat stress on *Macrophomina phaseolina* infection and DRR disease severity in ICC 4958 and JG62 chickpea genotypes. The pictures represent plants exposed to different treatments. Chickpea plants of ICC 4958 and JG 62 genotypes grown in pots were exposed to the individual and combined drought, heat, pathogen stresses to study the effect of the different abiotic stress treatments on DRR infection. The observations were made at 30 DAS.

Supplementary Figure S18.

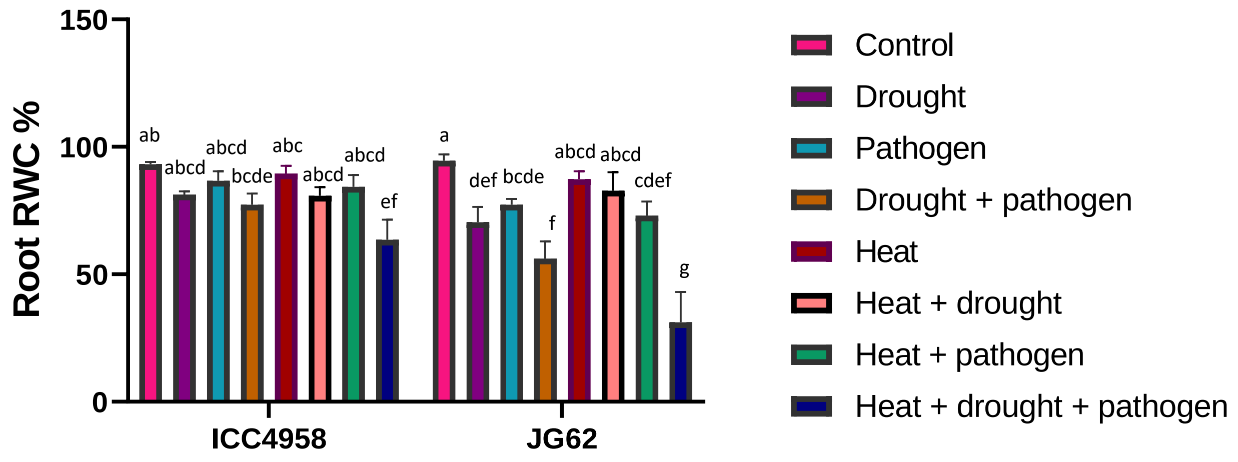

Supplementary Figure S18. **Effect of individual and combined drought, heat and pathogen infection on root water content of ICC 4958 and JG 62 plants pot grown under controlled conditions.** Plants were subjected to combined stress as mentioned in Supplementary Figure S14. Root samples for RWC measurement were taken 6 days post combined stress treatment. Each bar for RWC in the graph is the average of three replicates with SEM indicated by the error bar. Statistical significance between means were checked by two way ANOVA and Tukey's Posthoc test. The different letters denotes a significant difference between mean at $p<0.05$.

Supplementary Figure S19.

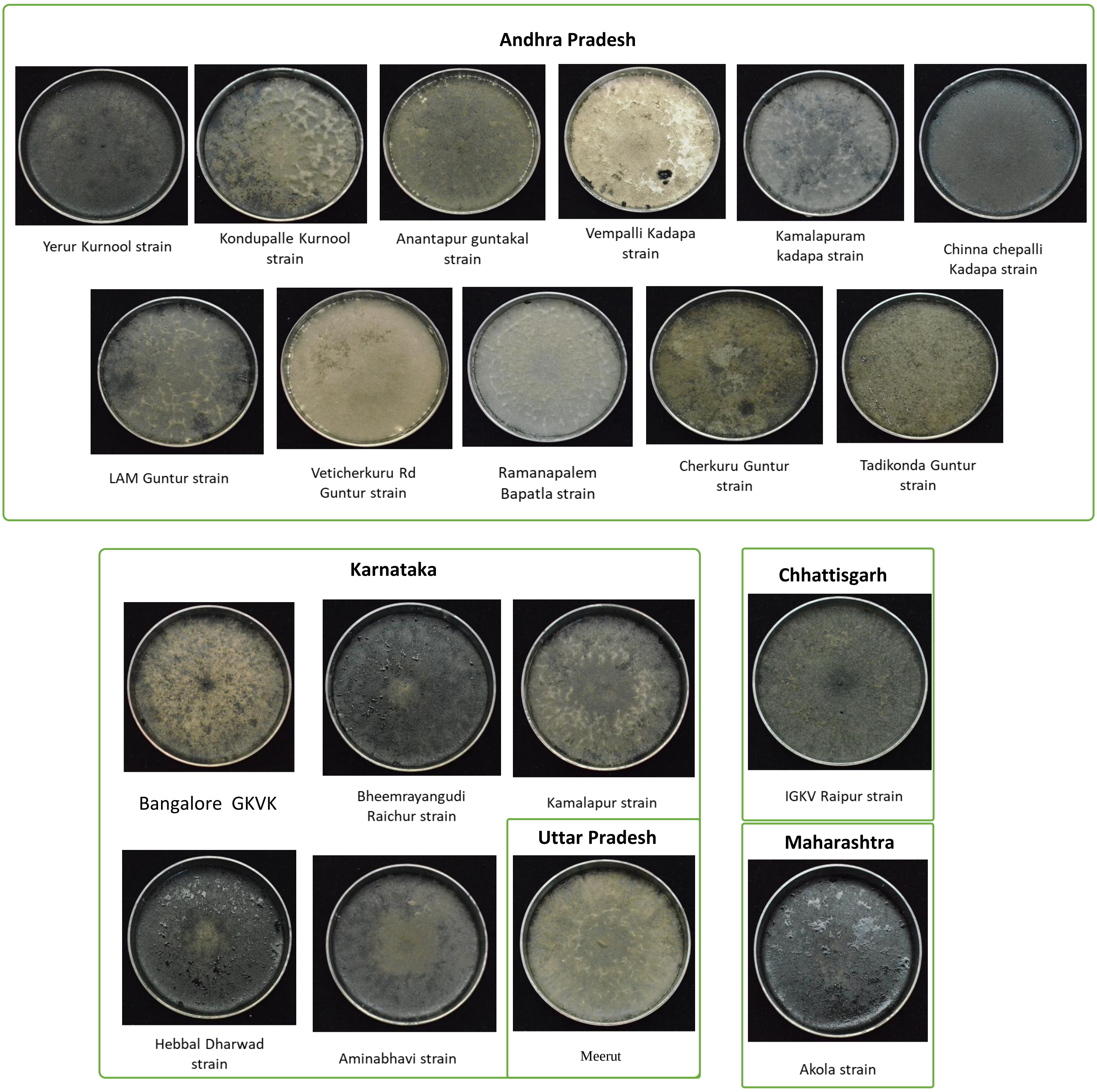

Supplementary Figure S19. Mycelial growth differences between the different strains of *Macrophomina phaseolina*, collected across different locations. The above images were captured from the isolates grown on PDA media over 10 days post-inoculation.

Supplementary Figure S20.

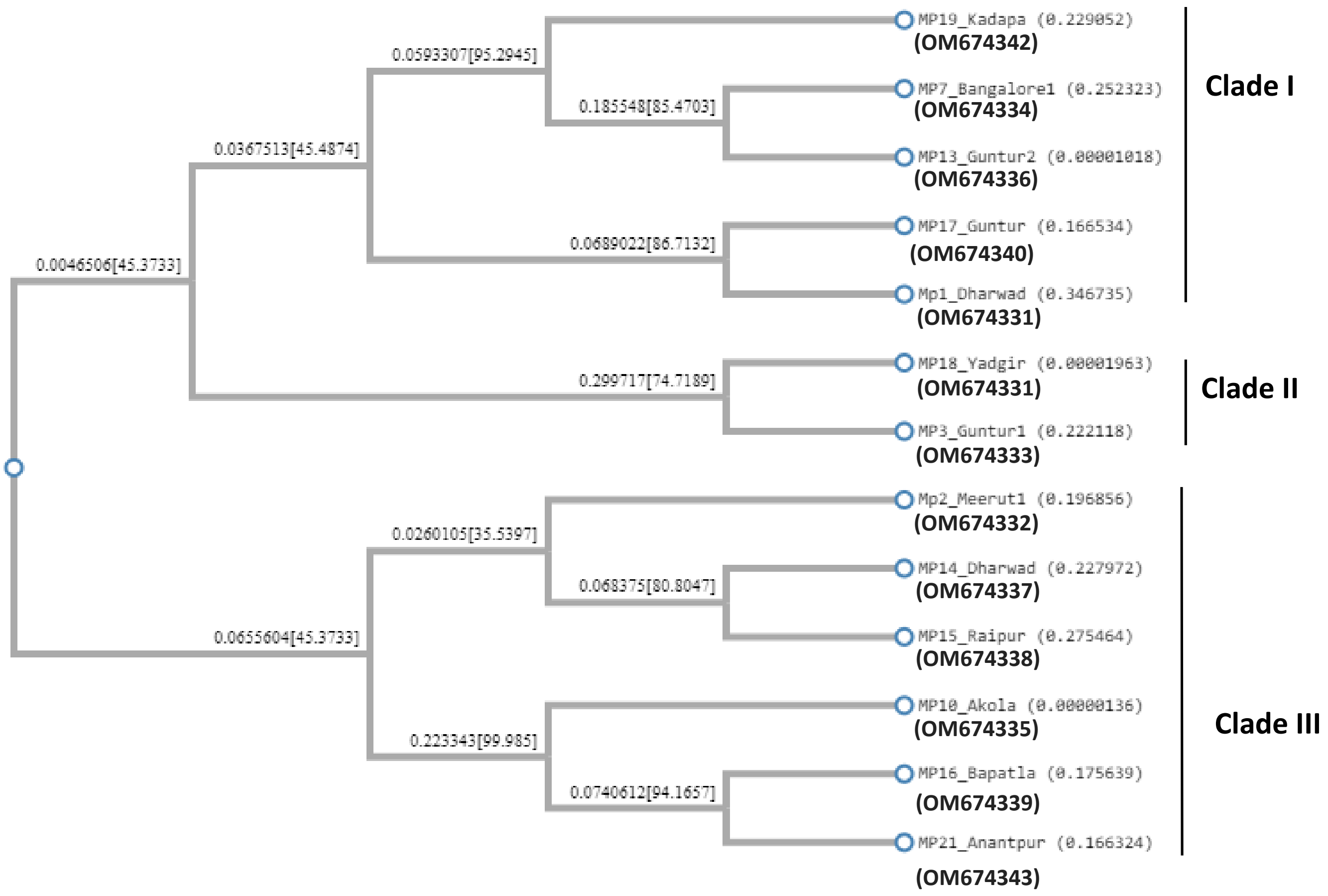

Supplementary Figure S20. Phylogenetic relationship between different strains of *Macrophomina phaseolina* collections made from surveys done in the period from 2017-21. Internal Transcribed Spacer Region amplified from their respective genomic DNA were used in the study. Alignment and phylogenetic reconstructions were performed using the function "build" of ETE3 v3.1.1 (Huerta-Cepas et al., 2016) as implemented on the GenomeNet (https://www.genome.jp/tools/ete/). ML tree was inferred using PhyML v20160115 ran with model and parameters: -o tlr --alpha e -f m --pinv e --nclasses 4 --bootstrap -2 (Guindon et al., 2010). Branch supports are the Chi2-based parametric values return by the approximate likelihood ratio test.

References

Huerta-Cepas J, Serra F, Bork P. ETE 3: Reconstruction, analysis, and visualization of phylogenomic data. *Mol Biol Evol.* 2016;33:1635–1638.

Guindon, S., Dufayard, J.F., Lefort, V., Anisimova, M., Hordijk, W. and Gascuel, O., 2010. New algorithms and methods to estimate maximum-likelihood phylogenies: assessing the performance of PhyML 3.0. Systematic biology, 59(3), pp.307-321.

Supplementary Figure S21.

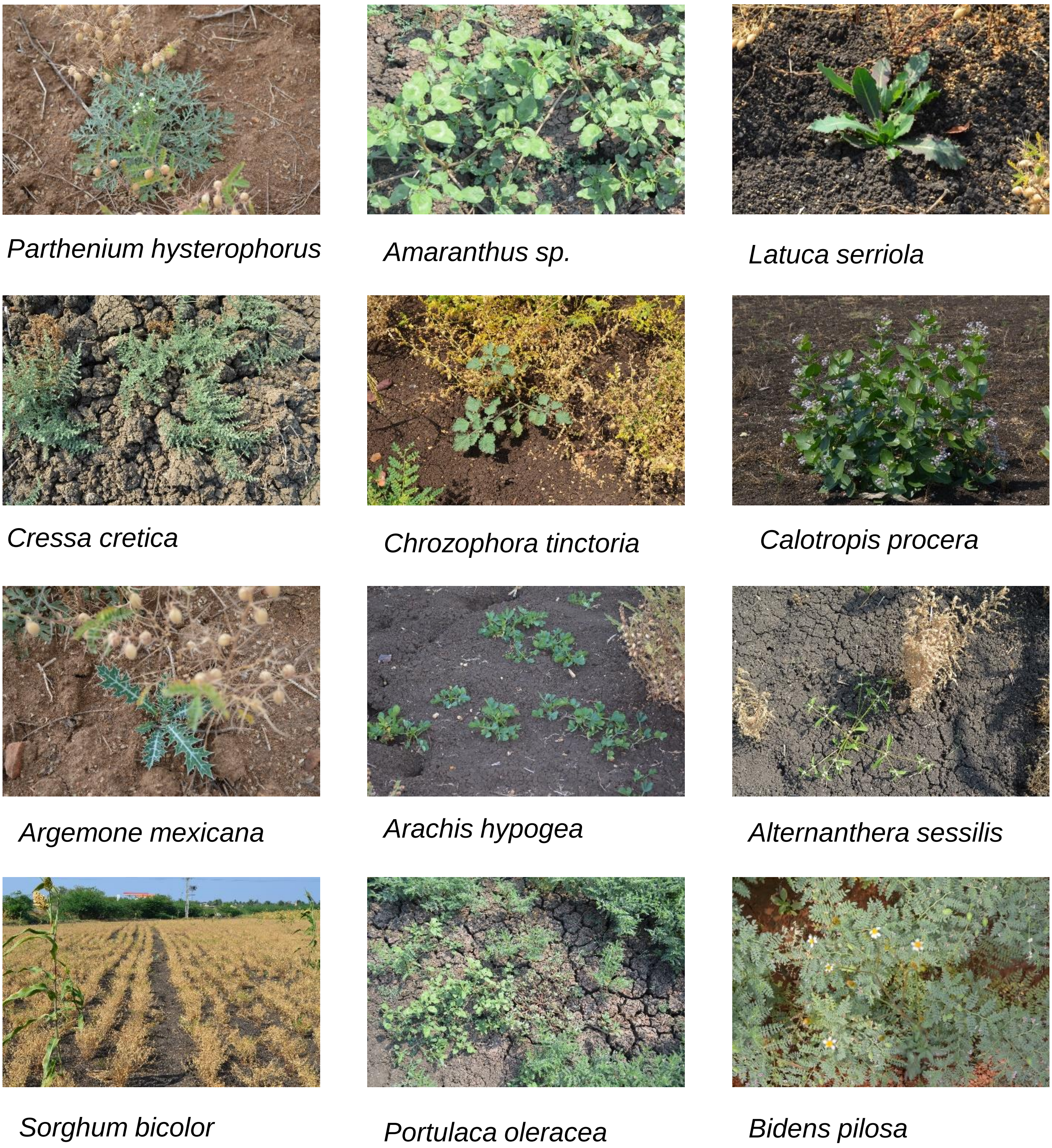

Supplementary Figure S21. Different weed species associated with the DRR infected fields. Plant images indicate the presence of different weed species that are found thriving in the dry root rot disease-infested chickpea fields (both experimental locations and farmers' fields in the current field trials). The observations indicate the possible non-host resistance against *M. phaseolina in* the weeds.
