# Supplementary material for: Combined Drought and Heat Stress Influences the Root Water Relation and Determine the Dry Root Rot Disease Development Under Field Conditions: A Study Using Contrasting Chickpea Genotypes
Source: Front Plant Sci. 2022 May 9;13:890551. doi: 10.3389/fpls.2022.890551 (PMC9128860; doi:10.3389/fpls.2022.890551)
Supplement: Supplementary File 1 — Raw data of all the figures and table presented in the manuscript. [file Data_Sheet_1.zip › Supplementary Tables.PDF]

Supplementary Information

Supplementary Tables 1-8

Combined drought and heat stress influences the root water relation and determine the dry root rot disease development under field conditions: a study using contrasting chickpea genotypes

Authors:

Aswin Reddy Chilakala[1#], Komal Vitthalrao Mali[1#], Vadivelmurugan Irulappan[1], Basavanagouda S. Patil[2], Prachi Pandey[1], Krishnappa Rangappa[3], Venkategowda Ramegowda[4], M. Nagaraj Kumar[5], Puli Chandra Obul Reddy[6], Basavaiah Mohan-Raju[4] and Muthappa Senthil-Kumar[1*]

*Correspondence:

Muthappa Senthil-Kumar, National Institute of Plant Genome Research, Aruna Asaf Ali Marg, P.O. Box No. 10531, New Delhi 110067, India. Email: skmuthappa@nipgr.ac.in.

Supplementary Table S1: Passport data, and DUS (distinctness, uniformity, and stability) characteristics of genotypes ICC 4958 and JG 62.

S No.	Traits and other information	ICC 4958	JG 62
1	**Plant height (cm)**	40.6 ± 4.3	36.9 ± 4.2
2	**Plant spread (cm)**	24.4 ± 5.2	18.7 ± 7.3
3	**Number of branches per plant**	11.5 ± 2.8	8.3 ± 4.4
4	**Number of pods per plant**	34.0 ± 8.7	23.4 ± 5.6
5	**Yield per plant (g)**	10.8 ± 4.5	5.6 ± 2.8
6	**100 seed weight (g)**	34.4 ± 2.4	14.7 ± 0.8
7	**Days per maturity**	117.0 ± 3.7	103.0 ± 4.5
8	**Pedigree status**	Parent	Parent
9	**Stress tolerance/sensitivity**	Drought tolerant	DRR susceptible, salinity sensitive
10	**Genome sequence status**	Sequenced (SRX9466450)	Sequenced (SRX9956275)
11	**Recommended region for cultivation**	It is best suited to areas having moderate rainfall of 60-90 centimeters per annum	It is best for short drier growing seasons

Reference- http://oar.icrisat.org/540/1/PMD_33.pdf

Supplementary Table S2: Information on the soil and climatic conditions about different experimental locations across the country where field trials were conducted.

S. No.	Locations of trials	State	Location	Lat/Long	Average Temp*	Average Rainfall*	Soil Type
**1**	**Location 1**	New Delhi	NIPGR	28.61/77.20	25.2 °C	693 mm	Clay
**2**	**Location 2**	Uttar Pradesh	Meerut	15.45/75.00	24.3 °C	885 mm	Black
**3**	**Location 3**	Karnataka	Dharwad	12.97/77.59	23.6 °C	831 mm	Red loamy soil/Laterite soil
**4**	**Location 4**	Karnataka	Bangalore	28.98/77.70	24.7 °C	933 mm	Sandy Loam/Loam
**5**	**Location 5**	Meghalaya	Shillong	25°41/92°09	17.1 °C	3385 mm	Sandy to claye loam Alluvial soil
**6**	**Location 6**	Andhra Pradesh	Kadapa	14.54/78.59	29.2 °C	753 mm	Black
**7**	**Farmer field 1**	Chhattisgarh	Raipur	21.25/81.62	26.8 °C	1276 mm	Red/black Ferralitic soil
**8**	**Farmer field 2**	Karnataka	Raichur	16.2/77.35	28 °C	713mm	Sandy loam to Sandy clay Black

Supplementary Table S3: Details of the field trial experiments conducted at six locations.

Trail locations	Date of Sowing (dd/mm/yyyy)	Other pest/ diseases observed	Soil Moisture measurement method adopted.	Date of harvest (dd/mm/yyyy)
Location 1 (New Delhi)	17.10.2020	Insignificant	Lutron soil moisture probe	10.03.2021
Location 2 (Meerut)	22.11.2020	Insignificant	Regulated irrigation	5.04.2021
Location 3 (Dharwad)	19.12.2020	Insignificant	Regulated irrigation	30.03.2021
Location 4 (Bangalore)	19.12.2020	Insignificant	Soil moisture probe	01.04.2021
Location 5 (Shillong)	12.11.2020	Insignificant	Regulated irrigation	24.02.2021
Location 6 (Kadapa)	30.12.2020	Insignificant	Regulated irrigation	Crop loss due to heavy rains @ 40 DAS

Supplementary Table S4: **Details of various experiments and the parameters measured in the study**

Sl.no	Key Experiments	Replications	Treatments	Details of treatments			Parameters measured	Key findings
				Irrigation	Fungicide	Temperature		
1.	**Experiment 1**: On season multilocation field trials	1. Six locations. 2. A minimum of 2/3 blocks per treatment in each location.	Mild pathogen	Frequent irrigation (80% FC)	Yes	Ambient	Root relative water content (A minimum of 4 biological samples in each block per treatment), DI	-ICC 4958 was found to be significantly tolerant to DRR disease. -Root relative water was found to affect the disease incidence negatively.
			Mild pathogen + drought	Less frequent irrigation (50% FC)	Yes	Ambient		
			Severe pathogen	Frequent irrigation (80% FC)	No	Ambient		
			Severe pathogen + drought	Less frequent irrigation (50% FC)	No	Ambient		
2.	**Experiment 2:** Off-season field trial	1. Single location. 2. Four blocks per treatment.	Mild pathogen	Frequent irrigation (80% FC)	Yes	High	Root relative water content (a minimum of 4 biological replications in each block per treatment), DI	-DRR Disease increased under combined heat and drought stresses. - ICC 4958 was found to be susceptible to DRR under heat stress. i.e., Breakdown of resistance due to high-temperature stress.
			Mild pathogen +drought	Less frequent irrigation (50% FC)	Yes	High		
			Severe pathogen	Frequent irrigation (80% FC)	No	High		
			Severe pathogen + drought	Less frequent irrigation (50% FC)	No	High		
3.	**Experiment 3:** Controlled conditions/ sick pot	1. Ten pots with 5 plants each. 2. The experiment was repeated thrice.	Control	90% FC	Autoclaved soil rite, No fungicide	Ambient (22/10ºC)	Root water potential, leaf water potential (Three biological samples per treatment were used each time), Disease severity	-Heat disrupts the resistance of ICC 4958 to DRR -Negative correlation between leaf water potential and DI
			Drought	40-60% FC	Autoclaved soil rite, No fungicide treatment	Ambient (22/10ºC)		
			Pathogen only	90% FC	Artificial inoculation of M. phaseolina in soil	Ambient (22/10ºC)		
			Combined stress	40-60% FC	Artificial inoculation of M.	Ambient (22/10ºC)		

					phaseolina in soil			
			Heat	90% FC	Autoclaved soil rite, No fungicide treatment	37/25ºC, 5 DAS		
			Heat + Drought	40-60% FC	Autoclaved soil rite, No fungicide treatment	37/25ºC, 5 DAS		
			Heat +Pathogen	90% FC	Artificial inoculation of M. phaseolina in soil	37/25ºC, 5 DAS		
			Heat+ Drought+ Pathogen	40-60% FC	Artificial inoculation of M. phaseolina in soil	37/25ºC, 5 DAS		
4.	**Experiment 4:** Controlled conditions pot experiments with field soil	Three replications.	Mild pathogen	Frequent irrigation (80% FC)	Yes	Ambient	Root system architecture traits like root length, root area, root volume, etc. (Three biological samples per treatment)	Root traits varied between the two genotypes across different treatments.
			Mild pathogen + drought	Less frequent irrigation (50% FC)	Yes	Ambient		
			Severe pathogen	Frequent irrigation (80% FC)	No	Ambient		
			Severe pathogen + drought	Less frequent irrigation (50% FC)	No	Ambient		
5.	**Experiment 5:** Paper Blot	Four biological samples	Control	NA	No infection	Ambient	Micro-sclerotia attachment using SEM	ICC 4958 was found to have a significantly lower percent of microsclerotia attached in comparison with JG 62.
			Pathogen infection		Seedings infected with microsclerotia	Ambient		

Supplementary Table S5: Details of the soil characteristics and nutrient content for the six experimental locations. Soil sampling was done at rhizosphere of chickpea (30-50 cm) from six different sites across each trail location. The data represented here is a mean of five replicates of soil sample test report.

Parameters	Location 1	Location 2	Location 3	Location 4	Location 5	Location 6
**pH**	7.18±0.32	7.09±0.27	5.77±0.34	7.11±0.22	4.71±0.04	6.88±0.27
**EC**	1.41±0.72	0.2±0.03	0.18±0.05	0.34±0.09	0.28±0.14	0.29±0.09
**OC**	0.51±0.38	0.62±0.08	0.71±0.11	0.29±0.01	1.36±0.67	0.52±0.04
**P**	88.2±65.72	36.83±0.58	21.07±5.62	20.15±9.93	9.02±4.8	57.03±4.24
**K**	774.33±564.27	234.67±6.51	230±15.39	133.67±4.51	212.33±23.97	394.67±32.15
**N**	125.3±75.22	149±106.02	188±0	118.33±12.42	377±21	279±32.08
**S**	133.67±32.02	5.01±3.72	8.7±1.78	12.9±1.23	1.28±0.23	7.69±3.67
**B**	0.53±0.26	0.72±0.05	0.55±0.31	0.19±0.02	0.43±0.08	0.32±0.05
**WHC**	37.87±2.32	45.6±1.56	30.13±1	30.87±1.59	50.97±7.57	41.97±8.4
**Sand**	40.8±2	36.13±4.16	52.8±2	61.47±11.72	26.2±5.52	54.8±2
**Silt**	40.93±1.01	15.33±1.15	9.33±1.15	17.6±9.52	45.93±2.25	12.67±2.31
**Clay**	15.6±4.61	48.53±4.62	37.87±1.15	20.93±4.39	27.87±4.16	32.53±1.15

Supplementary Table S6: Correlation table showing the relationship between different edaphic factors and disease incidence. The correlation analysis was performed using the disease incidence observed in pathogen treatments of locations 1, 2, and 3 with the soil characteristics in that region.

Correlations													
	**PDI**	**pH**	**EC**	**C (organic)**	**P**	**K**	**N**	**S**	**B**	**WHC**	**Sand**	**Silt**	**Clay**
**DI**	1												
**pH**	-0.538	1											
**EC**	-0.502	**.999***	1										
**C (organic)**	0.821	0.039	0.082	1									
**P**	-0.186	0.928	0.943	0.408	1								
**K**	-0.274	0.958	0.969	0.324	0.996	1							
**N**	0.993	-0.434	-0.395	0.883	-0.068	-0.157	1						
**S**	-0.461	0.996	**.999***	0.128	0.958	0.980	-0.352	1					
**B**	0.830	0.023	0.066	**1.000***	0.393	0.309	0.891	0.112	1				
**WHC**	0.923	-0.171	-0.129	0.978	0.207	0.118	0.962	-0.083	0.981	1			
**Sand**	-0.715	-0.205	-0.246	-0.986	-0.554	-0.477	-0.793	-0.291	-0.983	-0.929	1		
**Silt**	-0.484	**.998***	**1.000***	0.102	0.950	0.974	-0.376	**1.000***	0.086	-0.109	-0.266	1	
**Clay**	**0.969**	-0.730	-0.700	0.654	-0.424	-0.504	0.932	-0.667	0.666	0.798	-0.519	-0.685	1

*. Correlation is significant at the 0.05 level (2-tailed).

Supplementary Table S7: The list of pathogen collections showing different *Macrophomina phaseolina* strains across the country.

S No	Lab ID number	Name of the Location (Village, State name) with the Geographical coordinates	NCBI ID	Date of collection
0	SKMPt36	28.5308265,77.1645855<br>NIPGR, New Delhi, Delhi	MH509971.1	06.10.2017
1	SKMPt65	15.4847352,74.9752876<br>IARI RRS, Dharwad, Karnataka	OM674331	14.04.2020
2	SKMPt46	29.1417261,77.6825693<br>SVPUAT, Meerut, Uttar Pradesh	OM674332	16.06.2020
3	SKMPt67	16.2420450, 80.4479080<br>Vatticherukuru Rd, Guntur, Andhra Pradesh	OM674333	29.07.2021
4	SKMPt68	13.085556, 77.572167<br>GKVK, Bangalore, Karnataka	OM674334	29.07.2021
5	SKMPt69	20.7034744, 77.0246871<br>Akola, Maharashtra	OM674335	29.07.2021
6	SKMPt70	16.3886680, 80.4283270<br>Lam, Guntur, Andhra Pradesh	OM674336	29.07.2021
7	SKMPt71	14.3345154,75.6039816<br>Kamalapur, Dharwad, Karnataka	OM674337	29.07.2021
8	SKMPt72	21.235016, 81.7041701<br>IGKV, Raipur, Chhattisgarh	OM674338	20.04.2020
9	SKMPt73	15.986696, 80.381671<br>Cherukuru, Bapatla, Andhra Pradesh	OM674339	29.07.2021
10	SKMPt74	16.4149850, 80.4419570<br>Tadikonda, Guntur, Andhra Pradesh	OM674340	29.07.2021
11	SKMPt75	16.7323334,76.7989625<br>Bhimarayanagudi, Yadgir, Karnataka	OM674341	29.07.2021
12	SKMPt50	14.537813 78.560633<br>Vempalli, Kadappa, Andhra Pradesh.	OM674342	16.06.2020
13	SKMPt53	15.12121 77.353083<br>Guntakal, Anantapur, Andhra Pradesh.	OM674343	16.06.2020

Supplementary Table S8. Crops rotated with chickpea and weeds infesting chickpea fields

S. No.	Field Co-ordinates	City	DRR observed in chickpea	Species of weeds observed	Crops cultivated before chickpea	Crops after chickpea
**1**	12.9716° N, 77.5946° E	Bangalore	Yes	*Euphorbia hirta, Cyperus rotundus*	NA	NA
**2**	28.9845° N, 77.7064° E	Meerut	Yes	*Parthenium hysterophorus, Cyperus rotundus, Medicago sativa*	NA	NA
**3**	25°34' 43.583" N 91°53' 5.714E	Shillong	Yes	*Eupatorium sp.*	*Zea mays*	NA
**4**	15.4589° N, 75.0078° E	Dharwad	Yes	*Tridax procumbens, Amaranthus viridis*	*Arachis hypogea, Vigna radiata*	NA
**5**	16°12' 43.211" N, 77° 20'38.142" E.	Raichur	Yes	*Amaranthus viridis, Euphorbia hirta,*	NA	NA
**6**	21°15'4.98"N, 81°37'46.71"E	Raipur	Yes	*Amaranthus viridis, Parthenium hysterophorus*	NA	NA
**7**	28.6139° N, 77.2090° E	New Delhi	Yes	*Medicago sativa, Coriandrum sativum, Spinacea oleracea*	NA	NA
**8**	16.3067° N, 80.4365° E	Guntur	Yes	*Arachis hypogea, Cyperus rotundus, Tridax procumbens*	*Sorghum bicolor* (mixed crop)	NA
**9**	14.5171° N, 78.3679° E	Ganganapalle	Yes	*Euphorbia hirta, Mentha spp, Arachis hypogea*	NA	NA
